# Memory CD4 + T-Cells Expressing HLA-DR Contribute to HIV Persistence During Prolonged Antiretroviral Therapy

**DOI:** 10.3389/fmicb.2019.02214

**Published:** 2019-09-26

**Authors:** Eunok Lee, Peter Bacchetti, Jeffery Milush, Wei Shao, Eli Boritz, Daniel Douek, Remi Fromentin, Teri Liegler, Rebecca Hoh, Steve G. Deeks, Frederick M. Hecht, Nicolas Chomont, Sarah Palmer

**Affiliations:** ^1^Centre for Virus Research, The Westmead Institute for Medical Research, Sydney, NSW, Australia; ^2^Sydney Medical School, The University of Sydney, Sydney, NSW, Australia; ^3^Department of Epidemiology and Biostatistics, University of California, San Francisco, San Francisco, CA, United States; ^4^Department of Medicine, University of California, San Francisco, San Francisco, CA, United States; ^5^Leidos Biomedical Research, Inc., Frederick National Laboratory for Cancer Research, Frederick, MD, United States; ^6^Human Immunology Section, Vaccine Research Center, National Institutes of Allergy and Infectious Diseases, National Institute of Health, Bethesda, MD, United States; ^7^Centre de Recherche du CHUM et Département de Microbiologie, Infectiologie et Immunologie, Université de Montreal, Montreal, QC, Canada

**Keywords:** HLA-DR, CD4+ T-cells, prolonged ART, HIV persistence, cell activation/exhaustion markers, cellular proliferation, single-proviral sequencing

## Abstract

To date, most assays for measuring the human immunodeficiency virus (HIV-1) reservoir do not include memory CD4+ T-cells expressing the activation marker, human leukocyte antigen-antigen D related (HLA-DR). However, little is known concerning the role these cells play in maintaining persistent HIV-1 during effective antiretroviral therapy (ART). To address this issue, we examined, cellular activation/exhaustion markers (Ki67, CCR5, PD-1, Lag-3 and Tim-3) and viral gag-pol DNA sequences within HLA-DR− and HLA-DR+ memory CD4+ T-cell subsets longitudinally from the peripheral blood of six participants over 3 to ≥15 years of effective therapy. HLA-DR expression was readily detected during the study period in all participants. The average expression levels of CCR5, PD-1 and Tim-3 were higher on the HLA-DR+ T-cell subset whereas the average of LAG-3 expression was higher on their HLA-DR− counterpart. The proportion of HIV-infected cells increased within the HLA-DR+ subset by an average of 18% per year of ART whereas the frequency of infected HLA-DR− T-cells slightly decreased over time (5% per year). We observed that 20–33% of HIV-DNA sequences from the early time points were genetically identical to viral sequences from the last time point within the same cell subset during ART. This indicates that a fraction of proviruses persists within HLA-DR+ and HLA-DR− T-cell subsets during prolonged ART. Our HIV-DNA sequence analyses also revealed that cells transitioned between the HLA-DR+ and HLA-DR− phenotypes. The Ki67 expression, a marker for cellular proliferation, and the combined markers of Ki67/PD-1 averaged 19-fold and 22-fold higher on the HLA-DR+ T-cell subset compared to their HLA-DR− counterpart. Moreover, cellular proliferation, as reflected by the proportion of genetically identical HIV-DNA sequences, increased within both T-cell subsets over the study period; however, this increase was greater within the HLA-DR+ T-cells. Our research revealed that cellular transition and proliferation contribute to the persistence of HIV in HLA-DR+ and HLA-DR− T-cell subsets during prolonged therapy. As such, the HIV reservoir expands during effective ART when both the HLA-DR+ and HLA-DR− cell subsets are included, and therapeutic interventions aimed at reducing the HIV-1 reservoir should target HLA-DR+ and HLA-DR− T-cells.

## Introduction

Since 1996, antiretroviral therapy (ART) has dramatically reduced the mortality and morbidity caused by HIV infection (Palella et al., 1998). However, ART alone does not eradicate HIV. HIV-DNA persists as an integrated genome in long-lived or slowly dividing cells throughout therapy (Finzi et al., 1997; Siliciano and Siliciano, 2004); and viral replication resumes within a few weeks after the termination of ART (Chun et al., 2010; Hocqueloux et al., 2010).

Studies have shown that persistent HIV cellular reservoirs are established when an activated CD4+ T-cell becomes infected by HIV but transitions to a long-lived and quiescent memory T-cells instead of undergoing lytic infection (Finzi et al., 1997; Siliciano et al., 2003, 2007; Chomont et al., 2009; Shan et al., 2017). In addition, resting memory T cells can be infected directly by HIV (Swiggard et al., 2005; Agosto et al., 2007; Saleh et al., 2007; Dai et al., 2009). Many studies that investigate HIV persistence use CD4+ T-cells depleted for Human Leukocyte Antigen-antigen D Related (HLA-DR) as those cells that express HLA-DR are thought to be productively infected and short-lived (Chun et al., 1995). However, the degree to which the HLA-DR+ T-cells contribute to HIV persistence during effective therapy is unknown.

In treatment naïve HIV-infected individuals, the expression of HLA-DR on CD4+ memory T-cells is associated with HIV disease progression (Kestens et al., 1994). Recent studies have linked T-cell activation to HIV persistence including a study by Murray et al. (2014) which revealed that HIV-DNA persists in activated memory CD4 + T-cells (HLA-DR+) up to 1 year after therapy initiation (Chun et al., 2005). In addition, Cockerham et al. (2014) found that the most consistent association was between the frequencies of CD4 + or CD8 + T-cells expressing HLA-DR and HIV-DNA copy number as measured by digital droplet PCR in resting CD4 + T-cells. These observations suggest that HIV-DNA persists in memory CD4 + T-cells displaying an activated phenotype long after active viral replication is dramatically reduced by ART.

In order to fully define the role of CD4+ memory T-cells expressing HLA-DR in maintaining HIV persistence, we measured the levels of cellular activation and exhaustion markers over time in HIV-infected individuals who have been on ART for more than 15 years. By using single-proviral sequencing targeting the *gag-pol* region (p6 through nucleotides 1–900 of the gene encoding reverse transcriptase, p6-RT), we determined how these immunological markers are related to the frequency of HIV-infected T-cells. In addition, we investigated how these cellular markers are related to the genetic composition of HIV-DNA within HLA-DR− and HLA-DR+ CD4+ memory T-cell subsets during prolonged ART. Furthermore, we examined the persistence of HIV-infected HLA-DR+ memory T-cells and cellular transition between the HLA-DR+ and HLA-DR− cellular phenotypes by following HIV-DNA levels and viral DNA sequences longitudinally over 3 to ≥15 years of therapy.

Our study revealed that CD4+ memory T-cells that express HLA-DR are readily detected in both acute/early and chronic participants on prolonged therapy. Also, we found the proportion of HIV-infected HLA-DR+ T cells increases after prolonged therapy (≥15 years). Sequencing the HIV-1 genome revealed the same HIV viral sequences persisted over years of therapy in both the HLA-DR+ and HLA-DR− T-cell subsets. In addition, this sequence analysis showed some evidence that CD4+ memory T-cells have a capacity to change their cellular phenotypes between HLA-DR+ and HLA-DR− during ART. We observed that HLA-DR+ T-cells expressed higher levels of cellular activation/exhaustion and proliferation markers compared to their HLA-DR− counterpart. Therefore, our findings suggest that HIV persists in both HLA-DR+ and HLA-DR− CD4+ memory T-cell subsets and inclusion of both cell types should be considered when quantifying the viral reservoir and during the development of immune based treatment strategies.

## Materials and Methods

### Study Approval

This study was carried out in accordance with the recommendations of the institutional review board at the Western Sydney Health Department for the Westmead Institute for Medical Research (AU RED LNR/13/WMEAD/315), and the ethics review committees at the University of California San Francisco (UCSF) (10-01330/068192, 10-02631/083640) and Vaccine Gene Therapy Institute-Florida (VGTI-FL) (FWA 00004139). The protocol was approved by these committees. All study participants provided written informed consent in accordance with the Declaration of Helsinki.

### Participant and Clinical Samples

We included six HIV-1 subtype-B positive individuals on prolonged ART (> 15 years) from the SCOPE cohort in the study; 2 who initiated therapy during acute/early HIV infection (<6 months of infection before initiation of ART, AHI group) and 4 who initiated therapy during chronic HIV infection (>1 year of infection before initiation of ART, CHI group) (Supplementary Table 1). For five of these participants, peripheral blood was collected at 4 visits (Visit ID 1-4; after approximately 3, 5, 10, and 15 years of therapy) for this study. Participant 2518 provided peripheral blood during two visits (Visit ID 4 and 5, after approximately 15 and 17 years of therapy) (Supplementary Table 1). The viral load around the time of these visits was <40–75 copies (HIV-RNA) per ml. The CD4 counts were 462 cells/μL or greater during the study period.

### Sample Processing and Cell Sorting

The CD4 + T-cell subsets were sorted from frozen aliquots of peripheral blood mononuclear cells (PBMCs) stored for 4–16 years which contained 50–100 × 10^6^ cells that were collected from 5 of the participants at approximately years 3, 5, 10 on ART (Visit ID 1–3). At approximately 15 years of therapy, CD4+ T-cells were isolated from a leukapheresis for all 6 participants (Visit ID 4). Participant 2518 had a second leukapheresis sample collected for this study 2 years later at approximately 17 years on therapy (Visit ID 5). The cells were sorted using the following antibodies: CD3-Brilliant Violet 711 (clone OKT3, BioLegend), CD4-APC-eFluor 780 (clone OKT4, Thermo Fisher), CD14-V500 (clone M5E2, BD# 561391), LIVE/DEAD Aqua marker (Invitrogen# L34957), CD45RA-PECF594 (clone HI100, BD Biosciences), and HLA-DR− Brilliant Violet 421 (clone L243, BioLegend). CD3+CD4+ T-cells were gated on memory cells defined as CD45RA negative. HLA-DR± cells were then sorted on a BD FACS ARIA-II (Supplementary Figure 1). The HLD-DR± populations were sorted to >99% purity. Following sorting, the cell subsets were processed at 4°C and stored as a dry cell pellet at −80°C until analysis.

### Quantification of Immune Activation and Exhaustion Markers

Cryopreserved PBMC samples were thawed at 37°C and washed with Roswell Park Memorial Institute (RPMI) medium containing 10% heat inactivated fetal bovine serum, 1% L-glutamine, 1% sterile penicillin-streptomycin and 2 μg/mL DNase I (Sigma-Aldrich, St. Louis, MO, United States). PBMC were counted using Guava ViaCount Reagent on an BD Accuri flow cytometer (BD Bioscience). PBMC were plated in a 96-well v-bottom plate and stained for 15 min at 4°C, then washed twice with FACS buffer (phosphate-buffered saline containing 0.5% bovine serum albumin and 1 mM Ethylenediaminetetraacetic Acid). Finally, cells were fixed in 0.5% formaldehyde and data was acquired on a customized 4-laser BD LSR II Flow cytometer (BD Biosciences), with ≥200,000 lymphocytes collected for each sample. CPT beads (BD Bioscience) were used for instrument set up and target values were established using single peak medium range Rainbow Fluorescent Particles (Spherotech). BD^TM^ CompBead Plus Anti-Mouse Ig, ê beads or single stained PBMC were used for compensation controls. Data was compensated and analyzed in FlowJo V9 (TreeStar). A fluorescence minus one control was utilized to determine where to place the gate for PD- 1-, CD38- and HLA-DR− cells. We analyzed results as total CD4+ T-cells, divided out as naïve and memory based CD45RA expression and as CD4+ T-cells plus double negative T-cells (CD4-CD8- T-cells) in the event that CD4 was downregulated on the cell surface due to infection. The antibodies, fluorochrome conjugates and clones used for this analysis are included in the Supplementary Table 2. The cell viability of the frozen and leukapheresis samples used to measure the cellular activation/exhaustion markers ranged from 69.7–88.2% and 68.8–87%, respectively.

### Quantification of Intracellular HIV-DNA Levels

Intracellular HIV DNA levels within the sorted HLA-DR− and HLA-DR+ CD4+ memory T-cell subsets were measured using a previously described real-time RT-PCR method (Kumar et al., 2007). For this analysis, 96-12,186 HLA-DR− and HLA-DR+ memory T-cells per μL were used.

### DNA Extraction for Sequence Analyses

DNA was extracted from CD4+ HLA-DR− and HLA-DR+ memory T-cell subsets. For this extraction, 400 μL RNAzolRT (MRC, Inc.) was added to 1.5 mL Eppendorf tube containing the cell pellet. Next, 160 μL of sterile nuclease free water (Invitrogen) was added and mixed by inversion for 15 s followed by incubation for 15 min. The mixture was centrifuged at 16000 *g* for 15 min at room temperature. The top phase was removed and the bottom phase was used for DNA extraction. Nine hundred μL of DNAzol (MRC, Inc.) followed by 10 μL of glycogen (20 μg/μL, Qiagen) was added to the bottom phase. DNA was precipitated by adding 500 μL of 200 proof ethanol (Sigma-Aldrich). The mixture was incubated for 10 min at room temperature and centrifuged at full speed for 30 min. The supernatant was removed and the DNA pellet was washed with 75% ethanol twice. The pellet was air dried until no ethanol was visible. The pellet was dissolved in 300 μL of 8 mM NaOH (Sigma-Aldrich) followed by neutralization by adding 24 μL of 0.1 M HEPES (Gibco).

### Single-Proviral Sequencing (SPS)

Individual intracellular HIV-DNA sequences were obtained from the extracted DNA by single-proviral sequencing (SPS) that amplifies the *gag-pol* region of HIV genome (p6-RT) as previously described (Palmer et al., 2005; Kearney et al., 2008, 2009; Josefsson et al., 2012, 2013; von Stockenstrom et al., 2015). Briefly, the extracted DNA molecules were serially diluted (1:1-1:81) to endpoint dilution. Single HIV-DNA molecules were amplified using primers flanking the p6-RT region. We obtained single HIV-DNA molecules from approximately 206 to 200 × 10^3^ and 740 to 1.6 × 10^6^ HLA-DR+ and HLA-DR− memory T-cells, respectively (Supplementary Tables 3, 4). We obtained a total of 618 and 832 HIV-DNA sequences from the HLA-DR+ and HLA-DR− memory T-cell subsets, respectively. PCR amplification and sequencing of the HIV-DNA molecules at limiting dilution allowed us to determine the HIV infection frequency of the memory T-cells and also identify those HIV DNA sequences which were part of expansions of identical sequences (EIS) (Josefsson et al., 2013; von Stockenstrom et al., 2015).

### HIV-DNA Genetic Analysis

#### Inter-Participant Contamination Assessment

HIV-DNA p6-RT sequences derived from HLA-DR+ and HLA-DR− memory T-cell subsets were assessed for inter-participant contamination using a program for Maximum Likelihood based inference of large phylogenetic trees (Randomized Axelerated Maximum Likelihood; RAxML version 7.2.8.) with general time reversible and gamma distribution for the nucleotide model and rapid hill-climbing for the tree building algorithm (Stamatakis, 2006). The HIV sequences from the T-cell subsets and positive control for SPS were aligned using MAFFT (Katoh and Standley, 2013). The phylogenetic tree was annotated by ggtree in R (Yu et al., 2017). From this maximum likelihood phylogenetic tree, we found no evidence of cross-contamination between participant samples (Supplementary Figure 2).

#### Expansions of Identical HIV-DNA Sequences (EIS)

The expansions of identical HIV-DNA p6-RT sequences within HLA-DR− and HLA-DR+ memory T-cell subsets were identified within the CHI group using ElimDupes from the Los Alamos HIV sequence database^1^ with a pairwise genetic identity of 100%. HIV-DNA sequences from each participant were aligned using MAFFT (Katoh and Standley, 2013). For three participants from the CHI group, as the number of DNA sequences obtained from the frozen cell aliquots were at times limited, we analyzed the HIV-DNA sequences obtained from the 3 earlier visits together (Visit ID 1-3). The viral sequences from leukapheresis sample collected at the 4th visit were analyzed separately. For participant 2518, the viral sequences from visits 4 and 5 were independently analyzed for EIS. An EIS must contain at least two genetically identical HIV-DNA sequences derived from a specific CD4+ memory T-cell subset. However, if an HIV-DNA sequence from one of three earlier time points was genetically identical to a sequence from the later time point, these matching sequences were not considered a part of an EIS.

#### Identification of Genetically Defective HIV-DNA p6-RT Sequences

HIV-DNA p6-RT sequences containing G-A hypermutations were identified using Los Alamos Hypermut tool (Rose and Korber, 2000). The sequences containing premature stop codons, insertions/deletions causing a frameshift and/or internal deletions were identified by manual screening and the Los Alamos quality control tool (see footnote 1). All the defective sequences were included in the subsequent genetic analyses.

#### HIV-DNA Sequence Analysis

The longitudinal analysis of HIV genomic material allowed us to determine persistence of HIV within HLA-DR+ and HLA-DR− CD4+ memory T-cell subsets and investigate the amount of transition between these two cell types. For each participant, we constructed maximum likelihood phylogenetic trees using HIV-DNA sequences derived from the HLA-DR+ and HLA-DR− memory T-cell subsets during therapy using MEGA6 (1000 bootstrap replicates, general time reversible model with gamma distribution and proportion of invariant sites, gamma category 5) (Tamura et al., 2013). For 5 of the participants, we combined the HIV-DNA sequences obtained from 3 earlier time points (Visit ID 1–3) and compared these sequences to those obtained at the last time point (Visit ID 4). For the CHI participant, 2518, we compared the viral sequences obtained at 15.1 and 17.0 years on ART (Visit ID 4 and 5). To investigate whether the HIV genomes sequenced from the HLA-DR+ and HLA-DR− cells persist over time, we calculated the percentage of viral sequences from the earlier time points (visits 1–3 or for 2518, visit 4) that were genetically identical to at least one HIV-DNA sequence from the last time point (visit 4, or for 2518 visit 5) within the HLA-DR+ or HLA-DR− memory T-cell subsets. In addition, we investigated the transition between the HLA-DR+ and HLA-DR− cellular phenotypes using sequence analyses. In conducting these analyses we calculated the percentage of (HLA-DR+)-derived viral sequences from the earlier time points (visits 1–3 or for 2518, visit 4) that were genetically identical to at least one HIV-DNA sequence from the HLA-DR− T-cells at last time point (visit 4, or for 2518 visit 5). We also calculated the percentage of (HLA-DR−)-derived HIV-DNA sequences from earlier time points which were later found in the HLA-DR+ T-cell subset during ART. All the phylogenetic trees were annotated and visualized by R package, ggtree, and MEGA6 (Tamura et al., 2013; Yu et al., 2017, 2018).

### Statistics

We used restricted maximum likelihood to fit linear random-intercept-random-slope models to expression levels of HLA-DR, CCR5, LAG-3 and Tim-3 within HLA-DR+ and HLA-DR− CD4+ memory T-cell subsets at different time points during ART (Stata 15, StataCorp. 2017. Stata Statistical Software: Release 15. College Station, TX: StataCorp LLC). This method allows expression levels and their rates of change to differ in different participants. For proportions of HIV-infected T-cells in the HLA-DR+ and HLA-DR− subsets at longitudinal time points during ART, we used maximum likelihood statistical analysis modified from methods described previously (Josefsson et al., 2012, 2013; von Stockenstrom et al., 2015). We used the estimated model coefficients to derive estimated fold-differences of the proportions of infected cells and the expression of cellular activation/exhaustion markers between the HLA-DR+ and HLA-DR− cell subsets at various time points during therapy (Stata lincom command).

We defined dichotomous dependent variables for each HIV-DNA sequence being a part of an EIS and those sequences which indicate cellular persistence and transition. For these viral sequences, we applied mixed effects logistic regression with random person effects to obtain odds ratios to compare the last time point with the early time points during therapy, and to compare the HLA-DR+ to HLA-DR− T-cells.

## Results

### Persistent Expression of HLA-DR Within CD4+ Memory T-Cells During ART

**FIGURE 1:**
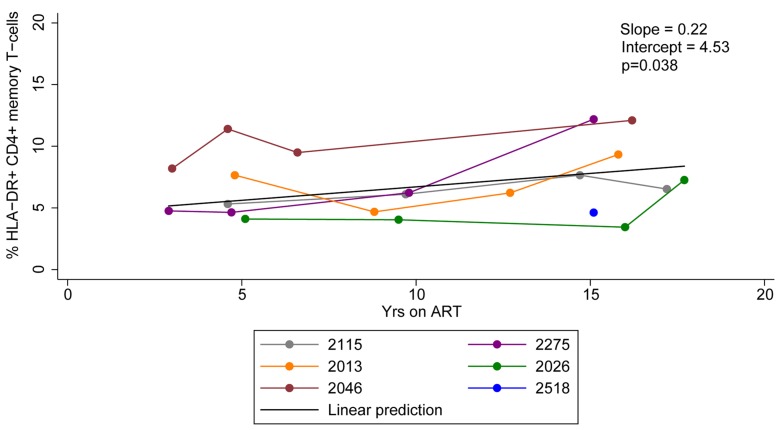
HLA-DR expression on CD4+ memory T-cells during ART. Proportions of CD4+ memory T-cells that express HLA-DR during ART were quantified. The linear prediction was performed using mixed effects regression. The slope indicates change of the proportion of HLA-DR+ CD4+ memory T-cells per year on ART and *p*-value (p) is for the slope.

We measured the expression of HLA-DR on memory CD4+ T-cells derived from HIV-infected individuals who initiated ART during acute/early and chronic infection at multiple time points during prolonged ART. The frequency of HLA-DR+ memory CD4+ T-cells ranged from 3.4 to 12.2% during 2.9–17.7 years of ART and increased by an average of 0.22% per year of therapy (95% CI = 0.02–0.42%, *p*-value = 0.038) (Figure 1). Our findings indicate HLA-DR+ memory CD4+ T-cells persist and even increase at a slow rate during therapy.

### Different Expression Profiles of Cellular Activation/Exhaustion Markers Within HLA-DR+ and HLA-DR− CD4+ Memory T-Cell Subsets During ART

**FIGURE 2:**
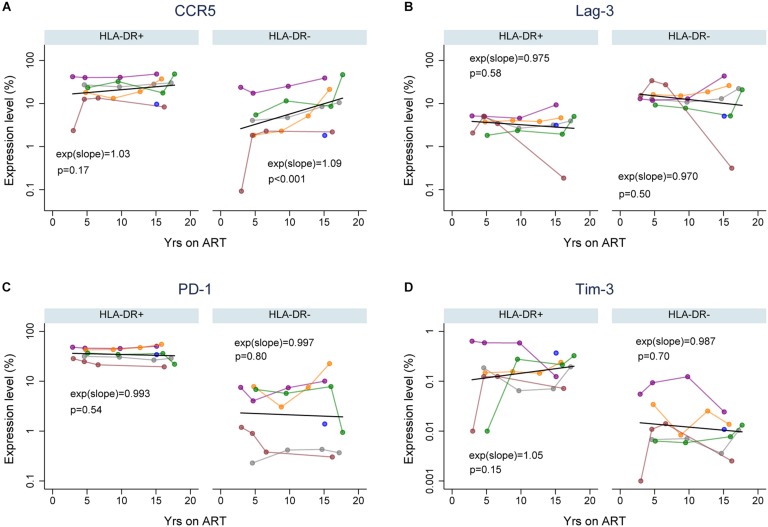
Expression of cellular activation/exhaustion markers on HLA-DR+ and HLA-DR– CD4+ memory T-cells during ART. Proportions of HLA-DR+ and HLA-DR– CD4+ memory T-cells that express CCR5 **(A)**, LAG-3 **(B)**, PD-1 **(C)**, and Tim-3 **(D)**. The colors of data points and lines indicate different participants; 2115 (gray), 2275 (purple), 2013 (orange), 2026 (green), 2046 (maroon), and 2518 (blue). A linear prediction was performed on the log transformed values using mixed-effects linear regression (black line). The changes of expression levels per year on therapy are indicated by exp(slope) (or *e*^*slope*^). An *e*^(slope)^ greater than 1 indicates an increasing rate and less than 1 indicates a decreasing rate of expression. The *p*-values (p) are derived by comparing the slopes to the null hypothesis of slope = 0, or *e*^(slope)^ = 1.

Immune exhaustion during chronic HIV infection is a process that causes T-cell dysfunction which is associated with high expression levels of immune checkpoint molecules (Kim and Ahmed, 2010; Khaitan and Unutmaz, 2011; Cox et al., 2017). However, the expression profile of cellular activation/exhaustion markers on HLA-DR+ CD4+ memory T-cells when HIV is suppressed by ART has not been characterized. Also, it is not clear whether the HLA-DR+ cellular compartment in an HIV-infected individual is characterized by T-cell dysfunction during ART. Therefore, we measured the frequency of HLA-DR+ and HLA-DR− memory CD4 + T cells expressing PD-1, LAG-3 and Tim-3 (markers for immune exhaustion, Cox et al., 2017); and CCR5 (virus-mediated pathogenesis and T-cell co-stimulation; Contento et al., 2008; Portales et al., 2012; Yang et al., 2012) by flow cytometry in samples from the six participants. We performed a linear prediction using a mixed effects regression model after performing a log transformation of percent T-cells expressing each of the cellular markers. The relative change in the frequency of the CD4+ memory T-cells expressing each cellular marker per year on therapy was determined by *e*^(slope)^ [exp(slope) in Figure 2], where the slope is estimated from the fitted linear line. For this calculation, an *e*^(slope)^ of greater than 1 indicates an increasing rate of expression and less than 1 indicates a decreasing rate of expression.

For each cellular marker, we estimated the fold-difference in their expression levels on HLA-DR+ versus HLA-DR− memory T-cells at 3, 5, 10, and 15 years of ART (calculated using the predicted linear line for each marker; Figure 2 and Table 1). HLA-DR+ cells had a higher level of expression of all the cellular markers except LAG-3 compared to HLA-DR− cells during ART. For CCR5 expression, the greatest difference between the HLA-DR+ and HLA-DR− cells was observed at 3 years of ART (Fold-difference = 6.36, 95% CI = 3.76–10.77, *p* < 0.001), but the difference was reduced to 3.43-fold at 15 years of therapy (95% CI = 2.26–5.21, *p* < 0.001). For PD-1 and Tim-3, HLA-DR+ cells had a 2-fold higher expression of these markers compared to HLA-DR− cells at all time points (*p* < 0.001–0.14). However, the HLA-DR− T-cells had approximately 3-fold higher expression of LAG-3 than the HLA-DR+ cells throughout ART (*p* < 0.001). Overall, most of the cellular activation/exhaustion markers were associated with the HLA-DR+ cells whereas LAG-3 was highly expressed on the HLA-DR− cells during ART.

**TABLE 1:** Estimated fold-difference of expression levels of cellular activation/exhaustion markers between HLA-DR+ and HLA-DR− CD4+ memory T-cells.

**Cellular marker**	**Years on ART**	**Fold-difference (HLA-DR+ vs. HLA-DR−)**	**95% CI**		***p*-value**
			**Lower**	**Upper**	
CCR5	3	6.36	3.76	10.77	<0.001
	5	5.74	3.72	8.87	<0.001
	10	4.44	3.27	6.02	<0.001
	15	3.43	2.26	5.21	<0.001
LAG-3	3	0.368	0.240	0.565	<0.001
	5	0.372	0.261	0.530	<0.001
	10	0.382	0.298	0.489	<0.001
	15	0.391	0.279	0.550	<0.001
PD-1	3	1.97	1.63	2.38	<0.001
	5	1.95	1.67	2.28	<0.001
	10	1.91	1.72	2.13	<0.001
	15	1.88	1.62	2.18	<0.001
Tim-3	3	1.80	0.82	3.95	0.143
	5	2.03	1.06	3.89	0.032
	10	2.76	1.75	4.35	<0.001
	15	3.74	2.01	6.97	<0.001

We also investigated the rate of the cellular activation/exhaustion marker expression throughout the treatment period of 3 to ≥15 years. An average of 3% (*p* = 0.17) and 9% (*p* < 0.001) increase in CCR5 expression per year of therapy was observed for the HLA-DR+ and HLA-DR− T-cells, respectively (Figure 2A). The rate of increase over time in CCR5 expression appeared to be greater in HLA-DR−, but the difference in rates between the HLA-DR+ and HLA-DR− T-cells had a *p*-value of 0.10. Both T-cell subsets revealed a slight decrease in the expression of LAG-3 and PD-1 (0.3–3%, *p* = 0.50–0.80, Figures 2B,C). The decrease in LAG-3 and PD-1 expression over time was similar between the T-cell subsets. Tim-3 expression increased by 5% on HLA-DR+ cells with each additional year of therapy (*p* = 0.15) whereas the HLA-DR− cells had a decreasing expression of Tim-3 of 1.3% per year (Figure 2D). However, the difference in the rates of change for Tim-3 expression during ART between the T-cell subsets was not statistically significant (*p* = 0.19). Overall, the changes in the expression of these cellular activation/exhaustion markers per year on ART were not found to be significant except for the expression of CCR5 on the HLA-DR− subset.

### Persistence of HIV-Infected Cells Within HLA-DR + and HLA-DR− CD4 + Memory T-Cell Subsets During ART

**FIGURE 3:**
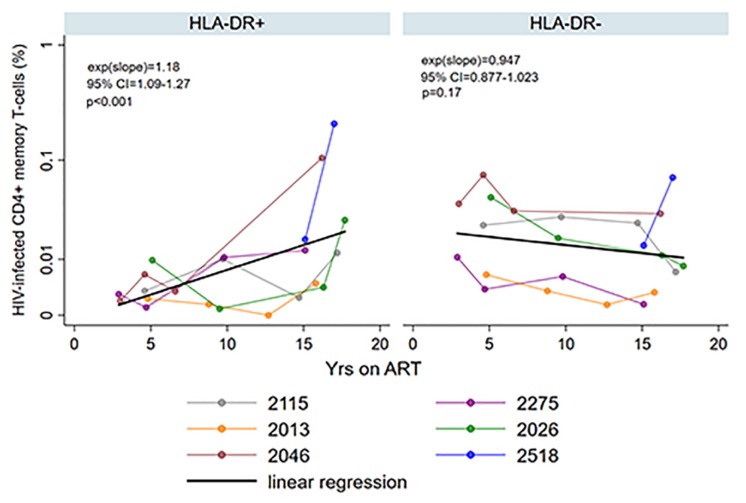
Persistence of HIV-infected HLA-DR+ and HLA-DR– CD4+ memory T-cells during ART. The proportion of HIV-infected T-cells (%) was estimated using maximum likelihood model derived from single-proviral sequencing. The change in the frequency of HIV-infected T-cells per year on ART [exp(slope)] was derived from mixed-effects negative binominal regression (black line). The 95% confidence intervals (95% CI) and *p*-value of the exp(slope) are also shown. The colors of the data points and the lines indicate different participants as shown in the legend.

HIV persistence within HLA-DR + CD4 + memory T-cells during prolonged ART is not clearly defined. We performed a previously described maximum likelihood method to estimate the number of cells infected within the HLA-DR+ and HLA-DR− CD4 + memory T-cell subsets in each participant at multiple time points during ART (Josefsson et al., 2012, 2013; Rosenbloom et al., 2015; von Stockenstrom et al., 2015). We employed multilevel mixed-effects negative binomial regression to estimate the change in the proportion of the infected T-cells per year on ART within the cell subsets. Using this model, we predicted the fold-difference of infection frequency between the HLA-DR+ and HLA-DR− subsets at 3, 5, 10, and 15 years of therapy. The change in the proportion of the infected T-cells is calculated by the e^(*slope*)^ of the predicted line [exp(slope) in Figure 3], where a value of <1 indicates a decrease and a value of >1 indicates an increase.

We observed an average of 18% increase in the proportion of infected HLA-DR+ T-cells during each additional year of therapy when participants from both the AHI and CHI groups were included (fold-change = 1.18/year, 95%CI = 1.09–1.27, *p* < 0.001) (Figure 3). However, the proportion of HIV-infected HLA-DR− T-cells decreased at an average rate of 5.3% per year of ART (fold-change = 0.947/year, 95% CI = 0.877–1.023, *p* = 0.17). During 3–10 years of ART, the proportion of the HIV-infected HLA-DR+ cells remained about 30–84% lower than the HLA-DR− counterpart (Table 2). After 15 years of treatment, however, the frequency of HLA-DR+ infected cells was 2-fold greater than the HLA-DR− cells (*p* = 0.013).

**TABLE 2:** Estimated fold-differences of proportions of HIV-infected cells within HLA-DR+ and HLA-DR− CD4+ memory T-cell subsets during ART.

**Years on ART**	**Fold-difference (HLA-DR+ vs. HLA-DR−)**	**95% CI**		***p*-value**
		**Lower**	**Upper**	
3	0.156	0.068	0.359	< 0.001
5	0.240	0.120	0.483	< 0.001
10	0.708	0.440	1.139	0.155
15	2.09	1.17	3.73	0.013

In addition, we quantified intracellular HIV-DNA levels by qPCR targeting the long terminal repeat (LTR) region of the HIV genome during ART (Kumar et al., 2007). Many of these measurements were below the limit of the assay (1 copy of HIV-DNA) due to the low numbers of T-cells that were used in the assay (Supplementary Table 5).

### Persistence of Viral p6-RT DNA Sequences Within HLA-DR+ and HLA-DR− CD4+ T-Cell Subsets During ART

**FIGURE 4:**
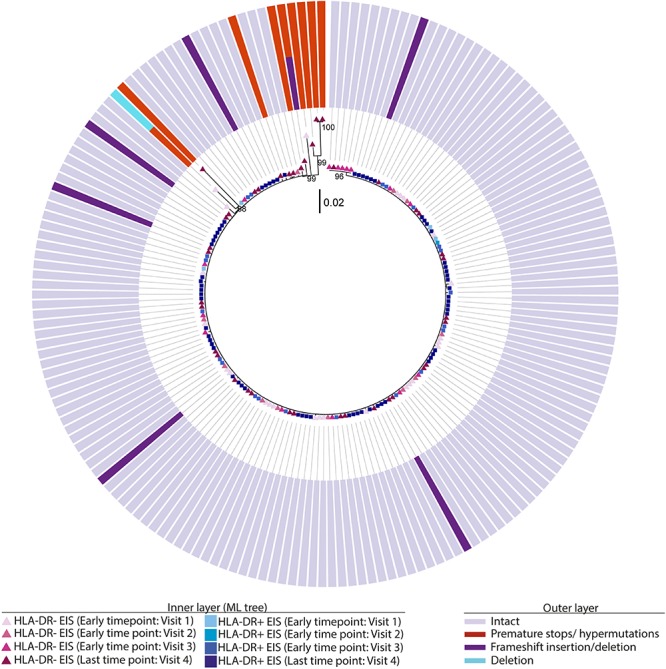
Intact and defective HIV-DNA p6-RT sequences within HLA-DR+ and HLA-DR– CD4+ memory T-cell subsets derived from an acute/early participant. A representative phylogenetic tree of a participant from the AHI group (2275). The inner layer shows the phylogenetic tree constructed by all HIV-DNA p6-RT sequences derived from the HLA-DR+ and HLA-DR– T-cells obtained from participant 2275 during therapy (Visits 1–4; 2.9–15.1 years after ART initiation). The outer layer shows defective genetic features for individual HIV-DNA sequences as shown in the legend.

Phylogenetic trees provide another way to visualize HIV persistence within the HLA-DR+ and HLA-DR− T-cell subsets. Therefore, we assessed the persistence of HIV-DNA p6-RT sequences within HLA-DR+ and HLA-DR− CD4+ memory T-cell subsets using phylogenetic analysis. The phylogenetic trees derived from the participants of the AHI group showed that HIV-DNA p6-RT sequences without stop codons within the HLA-DR+ and HLA-DR− T-cell subsets obtained during 2.9–17.2 years of therapy were genetically homogeneous (Figure 4 and Supplementary Figures 3–5).

**FIGURE 5:**
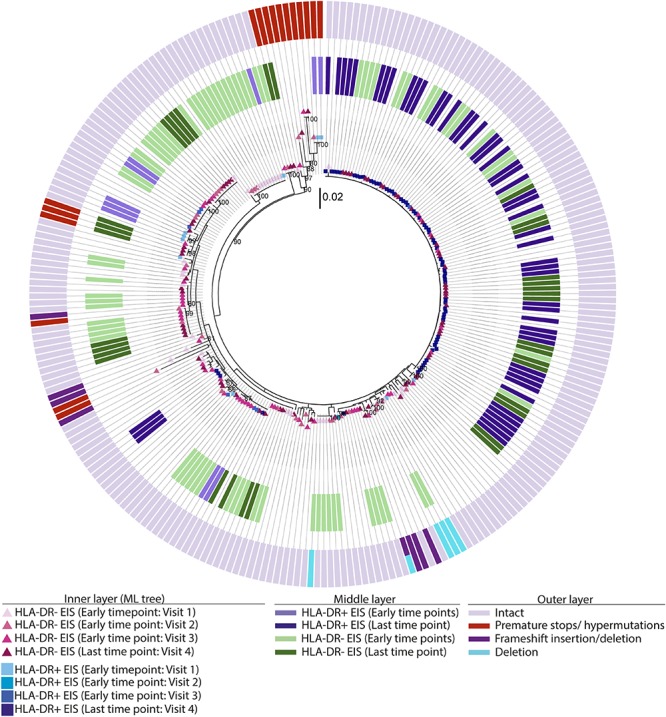
EIS, intact and defective HIV-DNA p6-RT sequences within HLA-DR+ and HLA-DR– CD4+ memory T-cell subsets derived from a chronic participant. A representative phylogenetic tree of a participant from the CHI group (2026). The inner layer shows a maximum likelihood phylogenetic tree that includes all HIV-DNA p6-RT sequences derived from the HLA-DR+ and HLA-DR– T-cells obtained from participant 2026 during therapy (CHI group). The middle layer shows individual HIV-DNA sequences which are part of an EIS within the HLA-DR+ and HLA-DR– T-cells obtained at three earlier time points (Visits 1–3; 5.1–16.0 years after ART initiation) and last time point (Visit 4; 17.7 years after ART initiation), The outer layer shows defective genetic features for individual viral sequences as shown in the legend.

**FIGURE 6:**
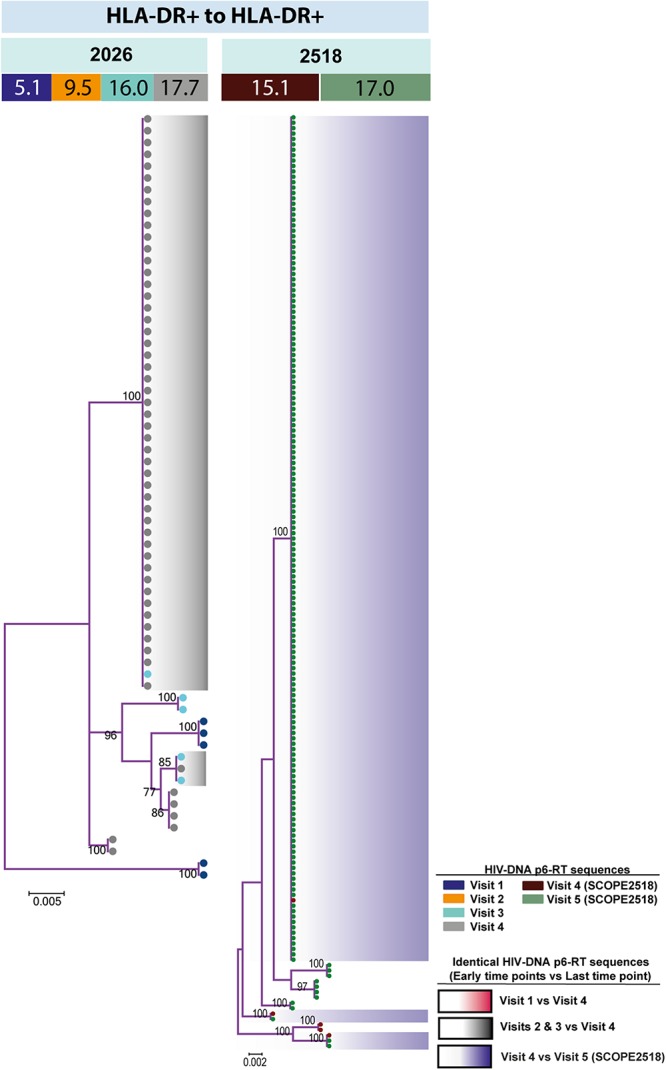
HIV-DNA p6-RT sequences persist within HLA-DR+ CD4+ memory T-cell subset during ART. Maximum likelihood phylogenetic trees using HIV-DNA sequences derived from monophyletic clades within the HLA-DR+ T-cell subset (HLA-DR+ to HLA-DR+). The trees were constructed for participants 2026 and 2518. The sampling time points (years after ART initiation) for individual HIV-DNA sequences are indicated under the participant IDs. The viral sequences derived from different sampling time points are shown with different colors that correspond to the visit IDs as shown in the legend. The shades indicate phylogenetic clades that contain identical HIV-DNA sequences derived from the three earlier time points and the last time point.

**FIGURE 7:**
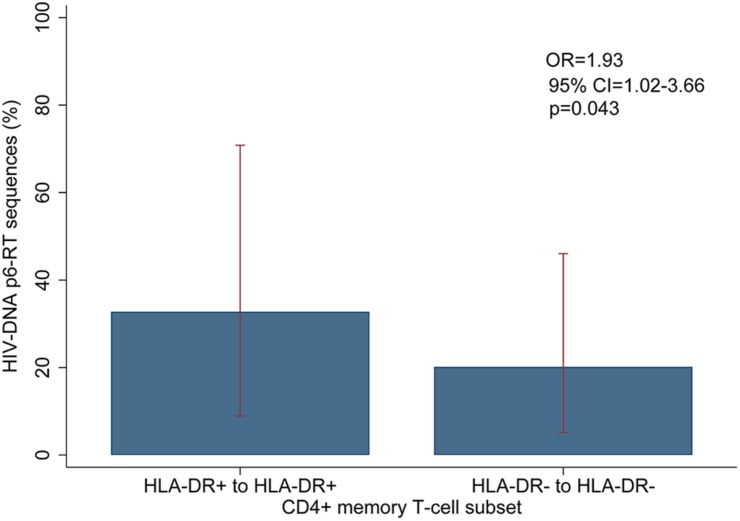
Overall comparison of the HIV-DNA p6-RT sequences that persist within the HLA-DR+ and HLA-DR– CD4+ T-cell subsets during ART. The percent of HIV-DNA sequences that persist within the HLA-DR+ and HLA-DR– T-cells are indicated as “HLA-DR+ to HLA-DR+” and “HLA-DR– to HLA-DR–”, respectively. The percent was estimated by fitting the mixed-effects logistic regression to all participants (blue bar). The 95% CIs of the percent values are shown as red capped lines. The odds ratio (OR) and its 95% CI and *p*-value compare “HLA-DR+ to HLA-DR+” to “HLA-DR– to HLA-DR–”.

For the participants from the CHI group, the HIV sequences derived from the HLA-DR+ T-cell subset at different time points during ART intermingled (Figure 5 and Supplementary Figures 6–8). For more accurate visualization of those HIV-DNA sequences that persisted over different time points during ART, we constructed phylogenetic trees using the viral sequences derived from monophyletic clades (Figure 6). For two participants (2026 and 2518), we found a total of six sequences that persisted over two years in the HLA-DR+ T-cell subset (Supplementary Table 6 and Figure 6). However, for all CHI participants, a total of 57 viral sequences derived from the HLA-DR− cell subset obtained at the earlier time points (3.0–10.0 years of ART; Visit ID 1-3) were genetically identical to sequences obtained at the last time point (15.8- years of ART; Visit ID 4; for 2518 this was compared from Visit ID 4 to Visit ID 5) (Supplementary Table 6 and Supplementary Figure 9). For all of the participants combined, the odds that an HIV-DNA sequence derived from one of the three earlier time points was identical to at least one sequence from the last time point during ART was approximately 2-fold higher within HLA-DR+ cell subset compared to the HLA-DR− (*p* = 0.043) (Figure 7). Within the HLA-DR+ cell subset, 33% of the HIV-DNA sequences at the earlier time points were identical to at least one viral sequence from the last time point. For the HLA-DR− cell subset, we found 20% of HIV-DNA sequences derived from the earlier time points were genetically identical to at least one viral sequence derived from the last time point.

### HIV-DNA Sequence Analysis Reveals a Fraction of CD4+ Memory T-Cells Change Between HLA-DR+ and HLA-DR− Phenotypes

Currently it is unclear as to the number of HIV-infected CD4+ memory T-cells transitioning between activated and resting cellular phenotypes. Therefore, we determined whether HLA-DR+ CD4+ memory T-cells containing HIV-DNA revert to the HLA-DR− phenotype during therapy. To do this, we calculated the number of HIV-DNA p6-RT sequences derived from the HLA-DR+ cell subset collected at the earlier time points that were genetically identical to the viral sequences derived from the HLA-DR− cell subset collected at the last time point from all of the participants (Supplementary Table 6). Also, we assessed whether the HLA-DR− memory T-cells transition to HLA-DR+ cellular phenotype by comparing the viral sequences from the HLA-DR− cell subset obtained at the earlier time points to those from the HLA-DR+ cell subset obtained at the last time point during ART (Supplementary Table 6).

**FIGURE 8:**
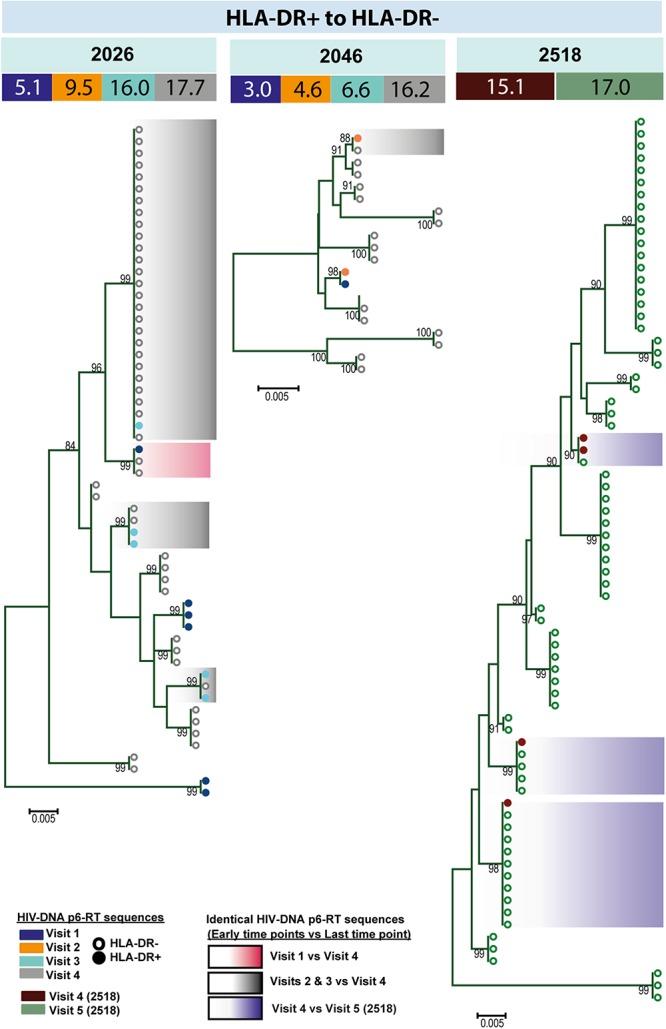
HIV-DNA p6-RT sequences from HLA-DR+ T-cells that are identical to sequences from HLA-DR– T-cells during ART. Maximum likelihood phylogenetic trees using HIV-DNA sequences derived from monophyletic clades that show sequences from the first time points of HLA-DR+ T-cells being identical to sequences from HLA-DR– T-cells at the last time point (HLA-DR+ to HLA-DR–). The trees were constructed for participants 2026, 2046, and 2518. The sampling time points (years after ART initiation) for individual HIV-DNA sequences are indicated under the participant IDs. The viral sequences derived from different sampling time points are shown with different colors that correspond to the visit IDs as shown in the legend. The shades indicate phylogenetic clades that contain identical HIV-DNA sequences derived from the HLA-DR+ T-cells obtained from the three earlier time points and the HLA-DR– T-cells from the last time point.

**FIGURE 9:**
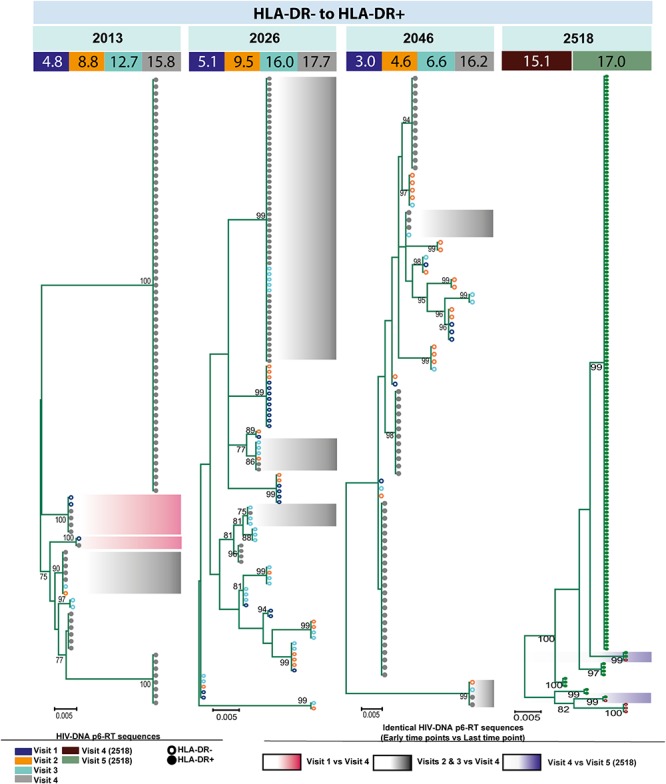
HIV-DNA p6-RT sequences from HLA-DR– T-cells that are identical to sequences from the HLA-DR+ T-cells during ART. Maximum likelihood phylogenetic trees using HIV-DNA sequences derived from monophyletic clades that show sequences from the first time points of HLA-DR– T-cells being identical to sequences from HLA-DR+ T-cells at the last time point (HLA-DR– to HLA-DR+). The trees were constructed for all CHI participants. The sampling time points (years after ART initiation) for individual HIV-DNA sequences are indicated under the participant IDs. The viral sequences derived from different sampling time points are shown with different colors that correspond to the visit IDs as shown in the legend. The shades indicate phylogenetic clades that contain identical HIV-DNA sequences derived from the HLA-DR– T-cells obtained from the three earlier time points and the HLA-DR+ T-cells from the last time point.

**FIGURE 10:**
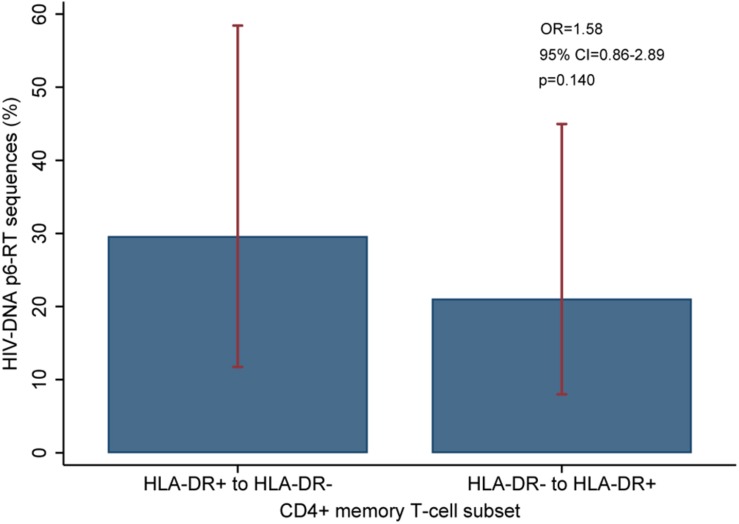
Overall comparison of the HIV-DNA p6-RT sequences that switch between HLA-DR+ and HLA-DR– CD4+ T-cell subsets during ART. The percent of viral sequences derived from the HLA-DR+ T-cell subset from the three early time points which were identical to at least one (HLA-DR–)-derived viral sequence from the last time point is indicated as “HLA-DR+ to HLA-DR–”. The percent of viral sequences derived from the HLA-DR– T-cell subset from the three early time points which were identical to at least one (HLA-DR+)-derived viral sequence from the last time point is indicated as “HLA-DR– to HLA-DR+”. The percent was estimated from the mixed-effects logistic regression fitted to all participants (blue bar). The 95% CIs of the percent values are shown as red capped lines. The odds ratio (OR) and its 95% CI and *p*-value compare “HLA-DR+ to HLA-DR–” versus “HLA-DR– to HLA-DR+”.

For three participants (2026, 2046, and 2518), we found a total of eight phylogenetic clades which contained genetically identical HIV-DNA sequences derived from the HLA-DR+ cell subset obtained at the three earlier time points and the HLA-DR− cell subset obtained at last time point (Figure 8). In all CHI participants, we found a total of ten clades that contained HIV sequences derived from the HLA-DR− cell subset obtained at the 3 earlier time points being genetically identical to viral sequences obtained from HLA-DR+ cell subset sorted at last time points (Figure 9). For all of the participants combined, the proportion of HIV-DNA sequences that indicate cellular transition from HLA-DR+ to HLA-DR− (30%) or from HLA-DR− to HLA-DR+ CD4+ memory T-cells (21%) was not statistically significant (OR = 1.58, 95% CI = 0.86–2.89, *p* = 0.14) (Figure 10).

### EIS Contribute to Proviral Persistence Within HLA-DR+ CD4+ Memory T-Cells During ART

Several studies have assessed the contribution of cellular proliferation to the persistence of HIV in different memory T cells by identifying and/or quantifying the number of genetically identical HIV-DNA sequences found in these cells at one time point or over time (Josefsson et al., 2013; Wagner et al., 2013; Imamichi et al., 2014; Kearney et al., 2015; von Stockenstrom et al., 2015; Simonetti et al., 2016; Hiener et al., 2017; Lee et al., 2017). However, many of these previous studies excluded HLA-DR expressing cells. We first determined the expression levels of the markers for cellular proliferation (Ki67 and the combined marker of Ki67/PD-1) on the HLA-DR+ and HLA-DR− CD4+ memory T-cells derived from the 4th visit during ART (Scholzen and Gerdes, 2000). We then compared these cellular expression levels with the genetically identical HIV-DNA p6-RT sequences obtained from the 4th visit during therapy. We also used a mixed effects logistic regression model to estimate changes in the frequency of genetically identical HIV-DNA p6-RT sequences during therapy within the HLA-DR+ and HLA-DR− CD4+ memory T-cell subsets derived from the CHI cohort, as these participants who initiated antiviral treatment during chronic infection contain genetically diverse intracellular viral DNA (Anderson et al., 2011; Josefsson et al., 2013; von Stockenstrom et al., 2015). In this study, we defined EIS as ≥2 genetically identical sequences which were derived from the same T-cell subset. For participants 2013, 2026, and 2046, we assessed the total number of sequences contributing to an EIS from the 3 earlier time points together (Visit ID 1-3) as these clinical samples were stored for 4–16 years and the number of cells sorted and the sequenced HIV genomes from these cells were at times quite low. We characterized the number of viral sequences contributing to an EIS from the samples obtained at the last time point separately, as the Visit ID 4 samples for all participants was a leukapheresis. For participant 2518, we characterized the number of sequences contributing to an EIS after 15.1 and 17.0 years of ART separately as the cells sorted at these time points (Visit ID 4 and 5) came from a leukapheresis.

To determine the proliferative nature of the HLA-DR+ and HLA-DR− CD4+ T-cells, we measured their expression of the cellular proliferation marker Ki67 alone or Ki67/PD-1 co-expression (Scholzen and Gerdes, 2000). The expression of these markers was determined for all 6 participants at Visit ID 4 (after approximately 15 years of therapy). We found that Ki67 expression averaged 19-fold higher on the HLA-DR+ cells (range = 17.3–29.7%) compared to the HLA-DR− cells (range = 0.829–2.63%) (*p* = 0.0005) (Supplementary Figure 10). Also, we the found Ki67/PD-1 co-expression level averaged approximately 22-fold higher on the HLA-DR+ cells (range = 6.11–18.3%) compared to the HLA-DR− cells (0.246–0.824) (*p* = 0.0008) (Supplementary Figure 10). For the CHI group, we did not find a strong positive correlation between the proportion of identical HIV-DNA sequences within their HLA-DR+ and HLA-DR− CD4+ memory T-cells and the level of Ki67 or Ki67/PD-1 expression on these cells at Visit ID 4 (Supplementary Table 7).

When the HIV-DNA p6-RT sequences were visualized by phylogenetic trees, we found that these EIS contain both intact and defective viral sequences (Figure 5 and Supplementary Figures 6–8). We also compared the number of HIV-DNA sequences from HLA-DR+ and HLA-DR− T-cell subsets contributing to an EIS at the earlier time points and the last time point during therapy. At the earlier time points during ART, the odds that an HIV-DNA sequence was part of an EIS within the HLA-DR+ or the HLA-DR− T-cell subset was similar (OR = 0.61–1.26, *p* = 0.20–0.82) (Supplementary Table 8). However, the odds that an HIV-DNA sequence contributes to an EIS in the HLA-DR+ T-cell subset was approximately 3 to 19-fold higher than their HLA-DR− counterpart at the last time point (*p* < 0.001). In addition, the proportion of HIV-DNA sequences derived from EIS was positively correlated with the proportion of HIV-infected CD4+ memory T-cells within the HLA-DR+ T-cell subset (ρ = 1.00, *p* = 0.083), whereas a negative correlation was observed within the HLA-DR− T-cells (ρ = −0.800, *p* = 0.20) (Supplementary Table 7).

**FIGURE 11:**
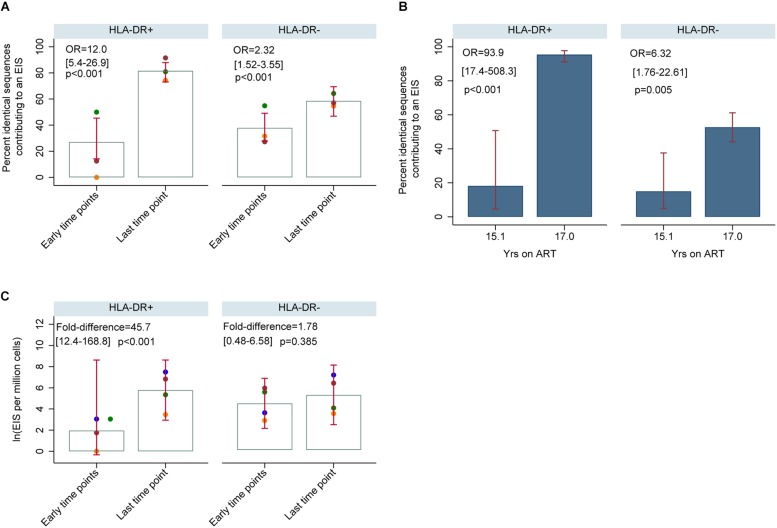
HIV-DNA p6-RT sequences derived from EIS within HLA-DR+ and HLA-DR– CD4+ memory T-cells during therapy (CHI participants). **(A)** Percent of HIV-DNA p6-RT sequences derived from EIS (% EIS) for participants 2013 (orange data points), 2026 (green data points) and 2046 (maroon data points). EIS was characterized using HIV-DNA sequences derived from the three earlier time points combined (“Early time points”) and the viral sequences derived from last time point (“Last time point”). The odds ratio (OR) compares “Last time point” to “Early time points.” The 95% CI (in square brackets) and the *p*-value (p) of the OR are also shown. The open gray bars indicate mean % EIS and the 95% CI of the mean is shown as the red capped lines. **(B)** Percent of HIV-DNA p6-RT sequences derived from EIS (% EIS) for participant 2518. The OR compares 17.0 to 15.1 years of ART. The 95% CI (in square brackets) and the *p*-value (p) are also shown. The filled blue bars indicate % EIS and its 95% CI is shown as the red capped lines. **(C)** Quantity of HIV-DNA sequences derived from EIS per million cells analyzed within the HLA-DR+ and HLA-DR– T-cell subsets. The gray open bars indicate mean of log transformed EIS per million cells and red capped lines indicate 95% CI of this mean. The fold-difference compares EIS per million cells of “Last time point” to “Early time points.” The 95% CI (in square brackets) and *p*-value (p) of the fold-difference are also shown. Different colors of the data points represent different participants; 2013 (orange), 2026 (green), 2046 (maroon), and 2518 (blue).

For participants 2013, 2026, and 2046, the number of genetically identical HIV-DNA p6-RT sequences within the HLA-DR+ T-cell subset was 4-fold greater at last time point (Visit ID 4) compared to three earlier time points combined (Visit ID 1-3) (Figure 11A). Within this HLA-DR+ cell subset, the odds of a viral sequence being a part of an EIS was 12-fold greater at the last time point compared to the earlier time points (95% CI = 5.4–26.9, *p* < 0.001). For the HLA-DR− cell subset, we found that the proportion of genetically identical HIV DNA sequences was only about 1.5-fold higher at the last time point compared to the earlier time points (OR = 2.32, 95% CI = 1.52–3.55, *p* < 0.001). For participant 2518, the proportion of the sequences derived from an EIS increased from 18 to 95% within the HLA-DR+ T-cells over two years of therapy (OR = 93.9, 95% CI = 17.4–508.3, *p* < 0.001) (Figure 11B). However, within the HLA-DR− T-cell subset, a smaller increase of genetically identical HIV sequences compared to the HLA-DR+ T-cell subset was observed (15–53%). Overall, for the CHI group, the number of viral sequences contributing to an EIS was 45.7 times greater within the HLA-DR+ T-cell subset obtained at the last time point compared to the earlier time points combined when the HIV-DNA sequences were normalized by the number of the HLA-DR+ cells we used in the sequencing (*p* < 0.001, Figure 11C). For the HLA-DR− T-cell subset, we found a 1.78-fold increase in the number of the viral sequences contributing to an EIS from the earlier time points compared to the last time point, however, this was not statistically significant.

## Discussion

HIV persists in resting memory CD4 T-cells during ART, but the contribution of CD4+ memory T cells expressing cellular markers of activation to the HIV reservoir is undefined. Understanding the contribution of CD4+ memory T-cells expressing HLA-DR to the persistent HIV reservoir is important for an accurate measurement of this HIV reservoir and would be crucial for the development of new immune based interventions. Here we performed a detailed longitudinal analysis of HIV persistence in HLA-DR+ and HLA-DR− memory T-cells sorted from peripheral blood of participants who initiated ART during acute/early and chronic infection. We also investigated the expression of cellular activation/exhaustion markers on these HLA-DR+ and HLA-DR− CD4+ memory T-cell subsets.

The assays currently used to detect inducible HIV proviruses commonly exclude CD4+ T-cells that express HLA-DR as these cells are believed to be actively transcribing HIV; thus, do not play an important role in HIV persistence during therapy (Chun et al., 1995; Siliciano and Siliciano, 2005). In our study, however, we found evidence for prolonged persistence of proviruses within HLA-DR+ CD4+ memory T-cell subset during therapy. First, the expression of HLA-DR+ on CD4+ memory T-cells and the proportion of HIV-infected HLA-DR+ T-cells increased with the duration of treatment. Second, for two participants treated during acute/early infection, the HIV-DNA p6-RT sequences derived from the HLA-DR+ T-cell subset over 2.9–17.2 years of ART were genetically homogeneous indicating stable infection of these cells from acute infection and throughout years of therapy. Lastly, we found two cases in the CHI group where HIV-DNA p6-RT sequences persisted within the HLA-DR+ T-cell subset over two years of therapy. Taken together our results suggest that we should consider including cells that express HLA-DR when measuring the HIV reservoir as HIV-infected HLA-DR+ T-cells do persist over years of therapy.

Studies have shown that HLA-DR expression correlates with the HIV-DNA level in CD4+ T-cells during 5.8–8.0 of therapy (Cockerham et al., 2014). In the current study, when participants were on therapy for 3 to ≥15 years, the proportion of HIV-infected HLA-DR+ T-cells increased at a rate of 18% per year. In the HLA-DR− memory T-cell subset, however, we found evidence for a decay in the proportion of the infected cells during ART at a rate of 5% per year, although it was not statistically significant. In addition, we measured HIV-DNA levels using qPCR targeting the LTR region, but many of these values were below the limit of the assay (1 HIV-DNA copy) due to the small numbers of cells assayed and therefore, were not informative. Nevertheless, our findings suggest a substantially slower decay rate of the HIV reservoir during therapy than previously reported (*t*_1__/__2_ = 43.9–44.2 months) when both the HLA-DR+ and HLA-DR− cell subsets are included (Finzi et al., 1999; Siliciano et al., 2003). We found that the frequency of HIV-infected cells increased within the HLA-DR+ T-cell subset during therapy, and this increase could compensate for the slow decrease of HIV-infected HLA-DR− T-cells.

Our genetic analysis of the HIV-DNA p6-RT genomic region suggests that two cellular mechanisms contribute to HIV persistence during therapy. First, over years of therapy, our HIV-DNA sequence analysis indicated that a fraction of the HLA-DR+ T-cells contribute to the persistent HIV reservoir of the HLA-DR− T-cell subset. For example, 30% of the HIV-DNA sequences from HLA-DR+ T-cells from the three earlier time points on ART were genetically identical to viral sequences derived from the HLA-DR− subset obtained at the last time point on ART. This suggests that a fraction of the HLA-DR+ T-cells transitioned to the HLA-DR− phenotype, downregulating their HLA-DR expression during therapy. We also found 21% of HIV-DNA sequences within the HLA-DR− subset identified at the three earlier time points were only found in the HLA-DR+ T cells at the last time point, indicating a cellular transition from a resting to activated (i.e., HLA-DR+) phenotype during therapy. Studies have shown that the HIV reservoir resides within an array of resting CD4+ memory T-cells with specific phenotypic markers during ART (Chomont et al., 2009; Perreau et al., 2013; Buzon et al., 2014). Also that effector and terminally differentiated effector CD4+ memory T-cell subsets commonly express HLA-DR (Corneau et al., 2017). Our genetic analysis suggests that a proportion of these differentiated CD4+ T-cell subsets cycle from HLA-DR− to HLA-DR+ phenotypes or vice versa during therapy and thus HLA-DR+ T-cells can contribute to the HIV reservoir in resting memory T-cells (Corneau et al., 2017).

In agreement with recent studies, we found cellular proliferation is the second cellular mechanism that contributes to HIV persistence during therapy (Josefsson et al., 2013; Wagner et al., 2013; Imamichi et al., 2014; Kearney et al., 2015; von Stockenstrom et al., 2015; Simonetti et al., 2016; Hiener et al., 2017; Lee et al., 2017). In our study, we found that genetically identical HIV-DNA p6-RT sequences increase during therapy within HLA-DR+ and HLA-DR− T-cells, indicating cellular proliferation continuously contributes to HIV persistence in both these cell subsets over years of ART. Also, we found a strong trend that the proportion of HIV-DNA sequences contributing to identical sequence clusters is positively correlated with the proportion of infected HLA-DR+ T-cells, but not within their HLA-DR− counterpart. We found that identical sequence expansions are comprised of either genetically intact or defective p6-RT HIV-DNA sequences. This indicates that cellular proliferation maintains the pool of HIV-infected cells containing genetically intact and defective p6-RT sequences, particularly within the HLA-DR+ T-cells.

Interestingly, we did not find a strong correlation between the proportion of HIV-DNA sequences belonging to a cluster of identical sequences and the markers for cellular proliferation (Ki67 and/or Ki67/PD-1 co-expression) within the HLA-DR+ and HLA-DR− T-cell subsets (Scholzen and Gerdes, 2000). This could be affected by the small number of CHI participants included in the correlation analysis. Also, this could be due to the fact that the expression of Ki67 and/or Ki67/PD-1 was determined within total CD4+ T-cells whereas identical sequence clusters were characterized within CD4+ memory T-cells. Cells expressing Ki67 are normally in the G_1_ phase which is before the division of a cell (Combadere et al., 2000). Perhaps the lack of correlation between Ki67 expression and clusters of identical sequences indicates that these identical sequences represent cells which have already proliferated whereas Ki67 expression represents those cells which have the capacity to proliferate.

The integration of the HIV genome occurs during the acute phase of infection when HIV is mainly M-tropic or non-syncytium-inducing and utilizes CCR5 for viral entry (Bjorndal et al., 1997; Berger et al., 1999; Rambaut et al., 2004; Colby et al., 2018). This indicates that CCR5-tropic proviruses are present within the HIV reservoir and can release CCR5-tropic virions when ART is interrupted. We found the frequency of CCR5 expression was greater on the HLA-DR+ T-cell subset at all time points during ART. These findings could indicate that the HLA-DR+ T-cell subset would be a major target for HIV-infection when HIV-infected individuals on long-term ART undergo treatment interruption.

We found Tim-3 expression was extremely low on both the HLA-DR+ and HLA-DR− CD4+ memory T-cell subsets compared to other cellular activation/exhaustion markers; however, it was highly associated with the HLA-DR+ T-cell subset during ART. In agreement with an earlier study, our finding indicates that Tim-3 expression can be observed on both activated and resting CD4+ T-cells (Corneau et al., 2017). A recent study showed that the CD4+ T-cell count and Tim-3 expression level are inversely correlated (Rallon et al., 2018). The low expression of Tim-3 within the HLA-DR+ and HLA-DR− T-cell subsets could be related to CD4+ T-cell restoration during therapy, as the participants we studied had near normal CD4+ T-cell counts during ART (Rallon et al., 2018).

Among the cellular activation/exhaustion markers we studied, the expression profiles of PD-1 and LAG-3 were particularly interesting, as it has been shown that CD4+ memory T-cells expressing these immune checkpoint molecules are highly enriched with inducible HIV proviruses (Fromentin et al., 2016). We found PD-1 expression was higher on the HLA-DR+ memory T-cell subset compared to their HLA-DR− counterpart during therapy. Conversely, we found LAG-3 expression was higher on the HLA-DR− T-cell subset when compared to the HLA-DR+ during ART. Together, our results indicate that both HLA-DR+ and HLA-DR− T-cell subsets express cellular activation/exhaustion markers which indicate they could contain inducible HIV proviruses.

The expression profiles of cellular activation/exhaustion markers suggest several therapeutic strategies that could reduce the persistent viral reservoir in these memory T-cells. First, the high level of CCR5 receptors on HLA-DR+ T-cells suggest that CCR5-antagonists such as maraviroc would be effective in reducing the HIV infection in these T-cells (Fatkenheuer et al., 2008; Rusconi et al., 2013). Second, the high frequency of PD-1 expression on HLA-DR+ T-cells provides a rationale for the use of immune checkpoint blockers as anti-latency agents to target these infected cells during ART. The effect of these anti-PD-1 agents would be greater against the HLA-DR+ T-cell subset compared to their HLA-DR− counterpart (Day et al., 2006; Teigler et al., 2017). However, anti-LAG-3 checkpoint blockers would be more effective against HLA-DR− T-cells, as these cells more frequently expressed LAG-3 compared to HLA-DR+ T-cells (Gandhi et al., 2006; Brignone et al., 2007, 2009). Overall, our study indicates that the immune checkpoint blockers could target different CD4+ memory T-cell subsets.

There are several limitations to our study, first we sequenced the p6-RT region of HIV not the full-length HIV genome, which could cause an over estimation of the HIV infection frequency and the identification of identical HIV-DNA sequences in both T-cell subsets (Hiener et al., 2017). Second, the long-term storage of some of the clinical samples limited the number of cells we could sort and our ability to sequence many HIV-DNA molecules from these cells, and this was especially true for HLA-DR+ T-cells. A longitudinal study using full-length HIV sequencing and integration site analysis from leukapheresis samples would be ideal to reconfirm our findings, as no sub-genomic viral region can accurately predict cellular proliferation (Ho et al., 2013; Maldarelli et al., 2014; Wagner et al., 2014; Cohn et al., 2015; Laskey et al., 2016; Hiener et al., 2017; Lee et al., 2017). However, such longitudinal assays are time consuming, costly and would also be limited by cell numbers that can be sorted from long-term frozen cell aliquots. Nonetheless, it is worth noting that the region we used for our genetic analysis (p6-RT) has been shown to be the best predictor for the full-length HIV genome and the clonality of this genome (clonal prediction score of 95) (Laskey et al., 2016). In addition, our cell sorting strategy excludes terminally differentiated effector memory CD4+ memory T-cells (T_*EMRA*_) which are a CD45RA positive cellular population as we focused on the memory T-cells which are CD45RA negative in this study. Due to the high expression of HLA-DR on T_*EMRA*_ cell subset, however, it is highly likely that this cellular subset can contribute to the persistence of CD4+ T-cells that express HLA-DR during prolonged ART (Corneau et al., 2017).

## Conclusion

Our study showed that although CD4+ HLA-DR+ memory T-cells express a high frequency of activation and exhaustion markers, this memory T-cell subset contributes to the persistent viral reservoir by cellular proliferation. Our sequence analyses provided evidence that the same HIV-DNA sequence can persist in HLA-DR+ T-cells for over two years of ART, indicating this T-cell subset has the potential to continually contribute to the persistent viral reservoir. Our research revealed cellular proliferation contributes to the persistence of HIV in HLA-DR+ and HLA-DR− T-cell subsets during prolonged therapy. Moreover, HIV sequence comparisons between early and late time points of ART also suggest a fraction of CD4+ memory T-cells can undergo cellular transition between HLA-DR+ and HLA-DR− cellular phenotypes. Importantly, both HLA-DR+ and HLA-DR− CD4+ memory T-cells should be included in future studies of the HIV reservoir, as these cells express critical immune checkpoint molecules that could be used for therapeutic strategies.

## Data Availability Statement

The datasets analyzed for this study can be found in the Genebank (accession numbers: MN234327 – MN235714).

## Ethics Statement

The studies involving human participants were reviewed and approved by this study was approved by the institutional review board at the Western Sydney Health Department for the Westmead Institute for Medical Research (AU RED LNR/13/WMEAD/315), and the ethics review committees at the University of California San Francisco (UCSF) (10-01330/068192, 10-02631/083640) and Vaccine Gene Therapy Institute-Florida (VGTI-FL) (FWA 00004139). All participants provided written informed consent prior to inclusion in the study. The patients/participants provided their written informed consent to participate in this study.

## Author Contributions

EL conducted SPS on the participant samples, analyzed the resulting data, and wrote the original manuscript. RH, SD, FH, JM, NC, and RF enrolled the participants, collected the tissues and/or sorted the cell subsets from the participant samples. EL, TL, and JM prepared the participant samples. EB and DD developed and assisted with the HIV-DNA extraction from cells of the participants. JM measured the cell activation/exhaustion markers and contributed to methods. TL performed the qPCR. WS designed the sequence alignment workflows. PB conducted the statistical analysis and contributed to review and editing of the results and methods. SP designed the study and supervised the work performed. All authors read and edited the manuscript.

## Conflict of Interest

WS was employed by company Leidos Biomedical Research, Inc. The remaining authors declare that the research was conducted in the absence of any commercial or financial relationships that could be construed as a potential conflict of interest.

## Footnotes

1 https://www.hiv.lanl.gov/
