# Supplementary material for: Memory CD4 + T-Cells Expressing HLA-DR Contribute to HIV Persistence During Prolonged Antiretroviral Therapy
Source: Front Microbiol. 2019 Sep 26;10:2214. doi: 10.3389/fmicb.2019.02214 (PMC6775493; doi:10.3389/fmicb.2019.02214)
Supplement: Supplementary file 1 [file Data_Sheet_1.PDF]

Supplementary Material

1 Supplementary Figures

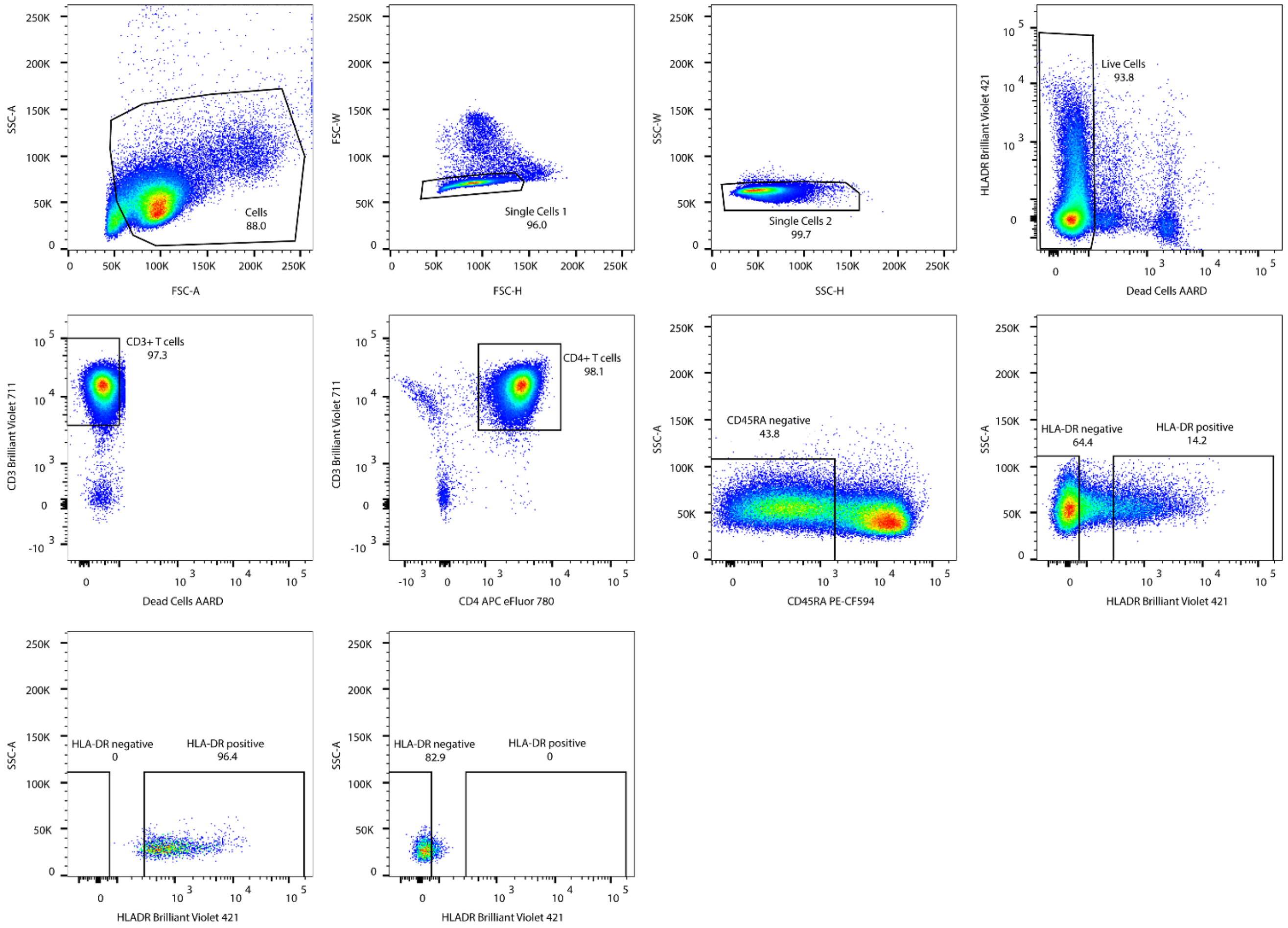

Supplementary Figure 1. Gating strategy for obtaining HLA-DR+ and HLA-DR- CD4+ memory T-cell subsets.

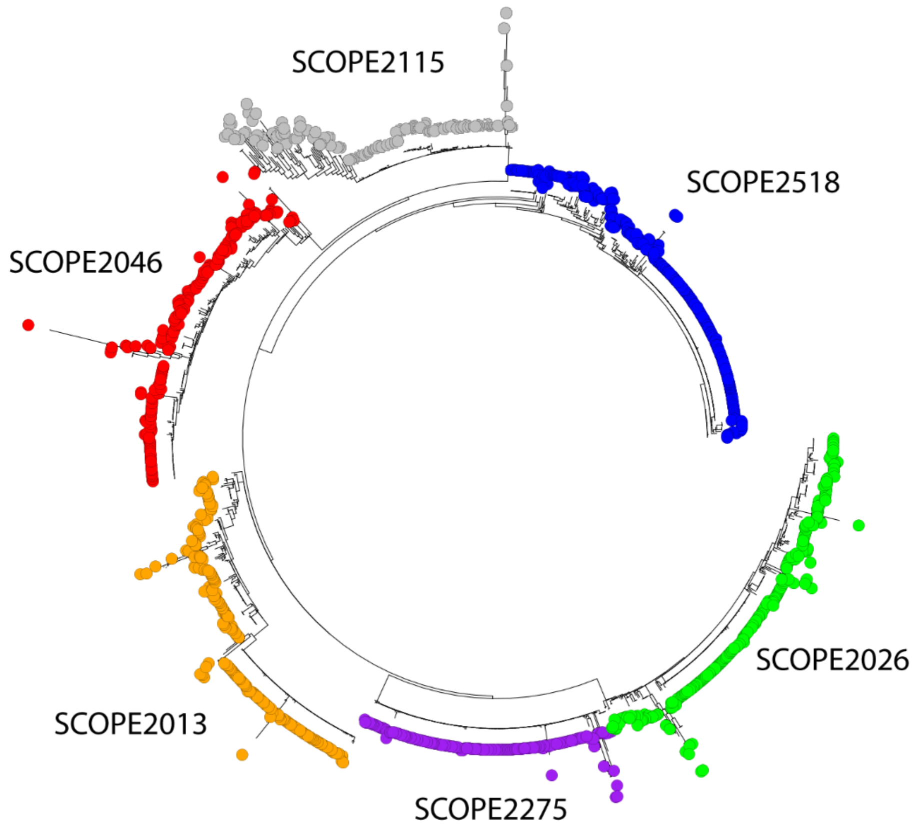

Supplementary figure 2. Maximum likelihood tree for interparticipant contamination assessment.

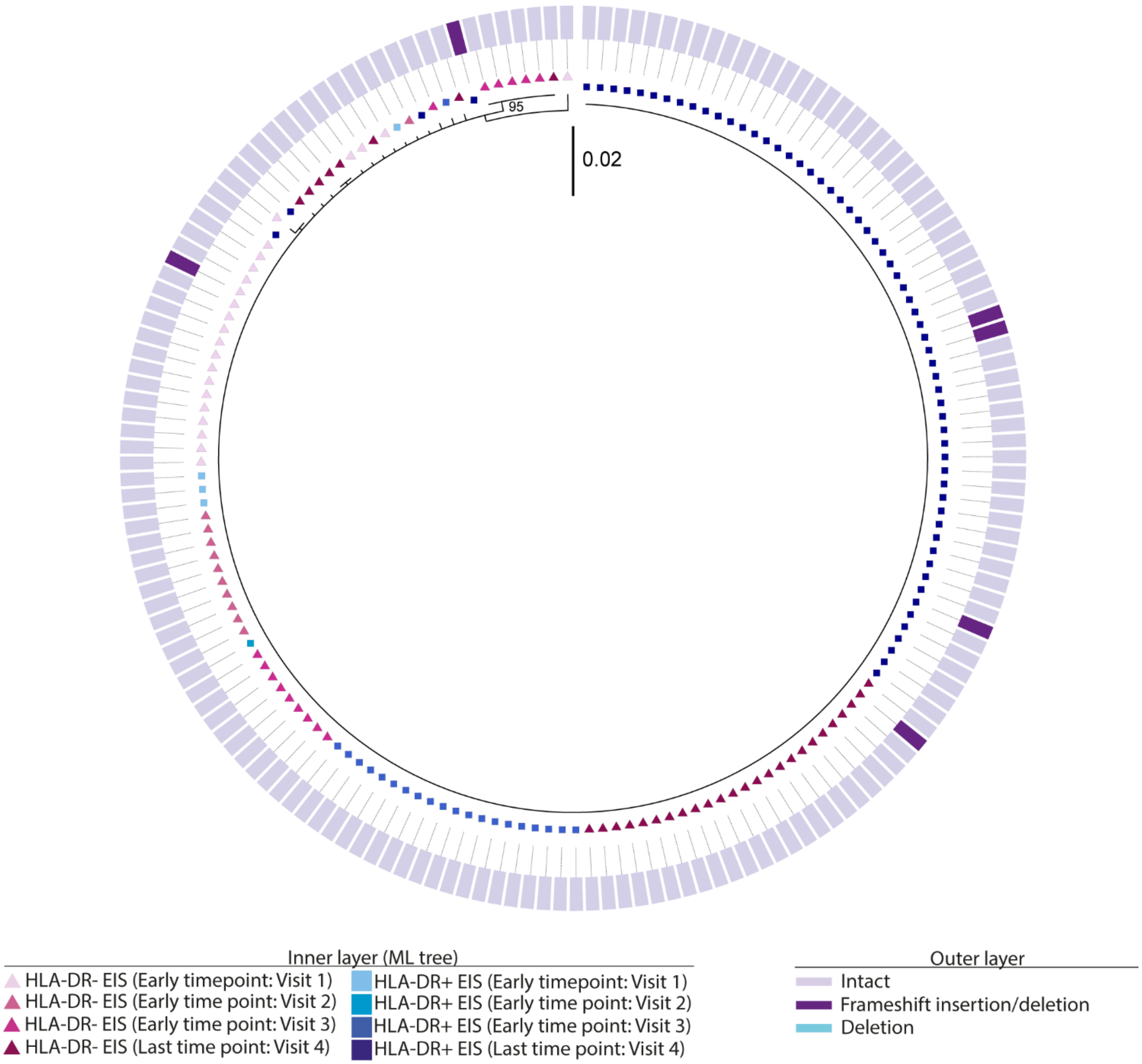

Supplementary figure 3. Maximum likelihood tree for participant 2275 (HIV-DNA p6-RT sequences with stop codons excluded). The inner layer shows the phylogenetic tree and the outer layer shows defective genetic features for individual HIV-DNA p6-RT sequences.

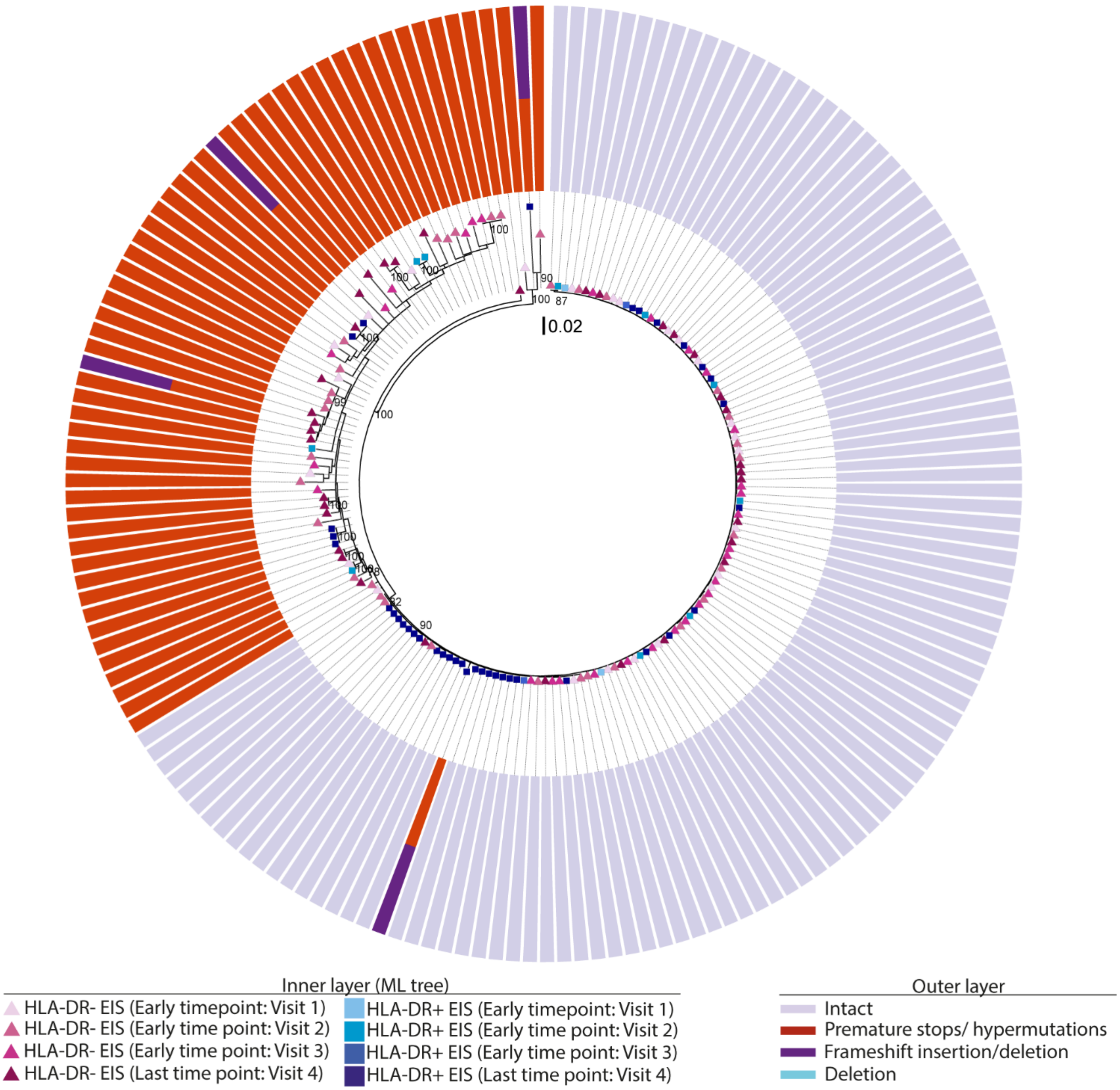

Supplementary figure 4. Maximum likelihood tree for participant 2115 (HIV-DNA p6-RT sequences with stop codons included). The inner layer shows the phylogenetic tree and the outer layer shows defective genetic features for individual HIV-DNA p6-RT sequences.

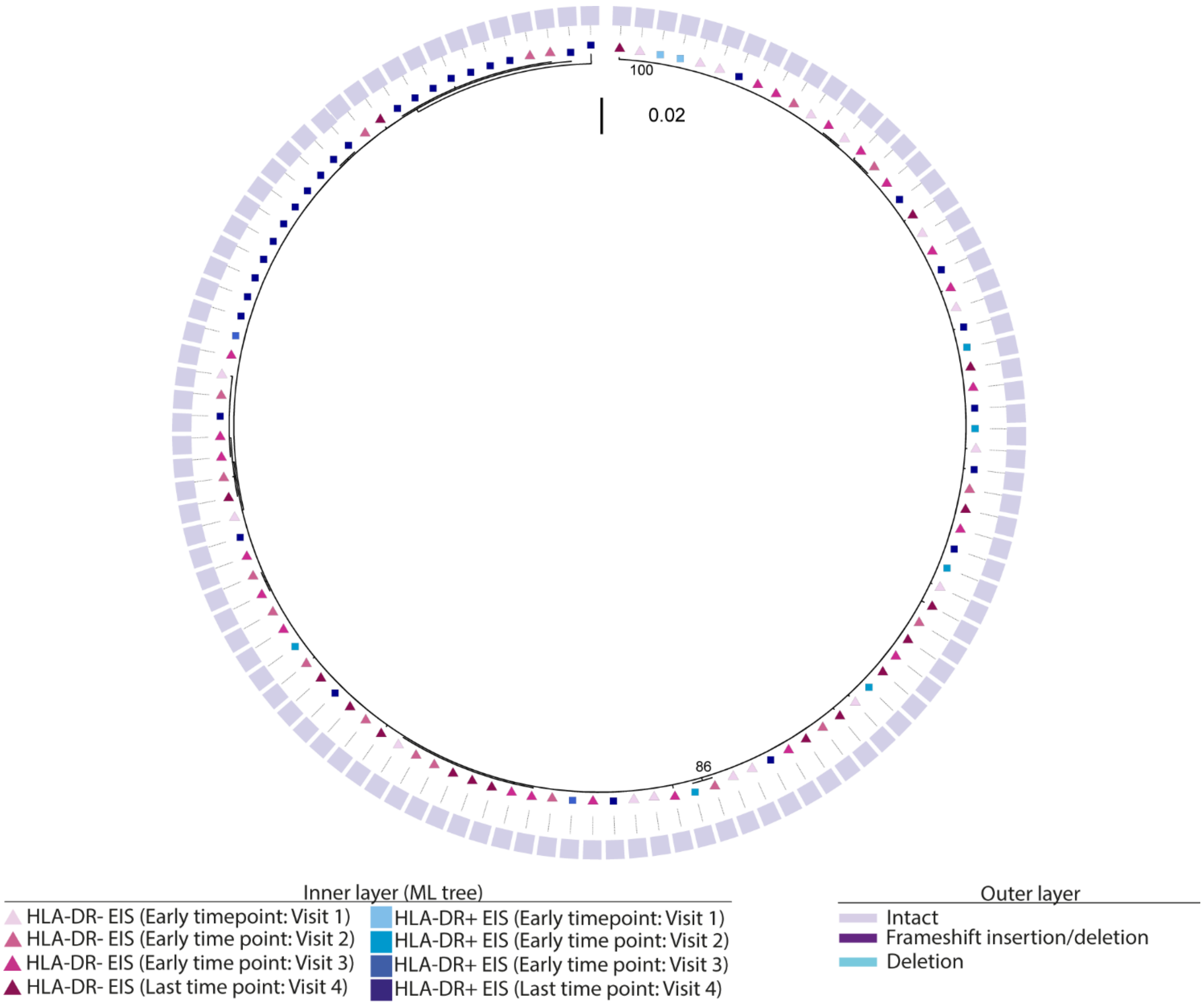

Supplementary figure 5. Maximum likelihood tree for participant 2115 (HIV-DNA p6-RT sequences with stop codons excluded). The inner layer shows the phylogenetic tree and the outer layer shows defective genetic features for individual HIV-DNA p6-RT sequences.

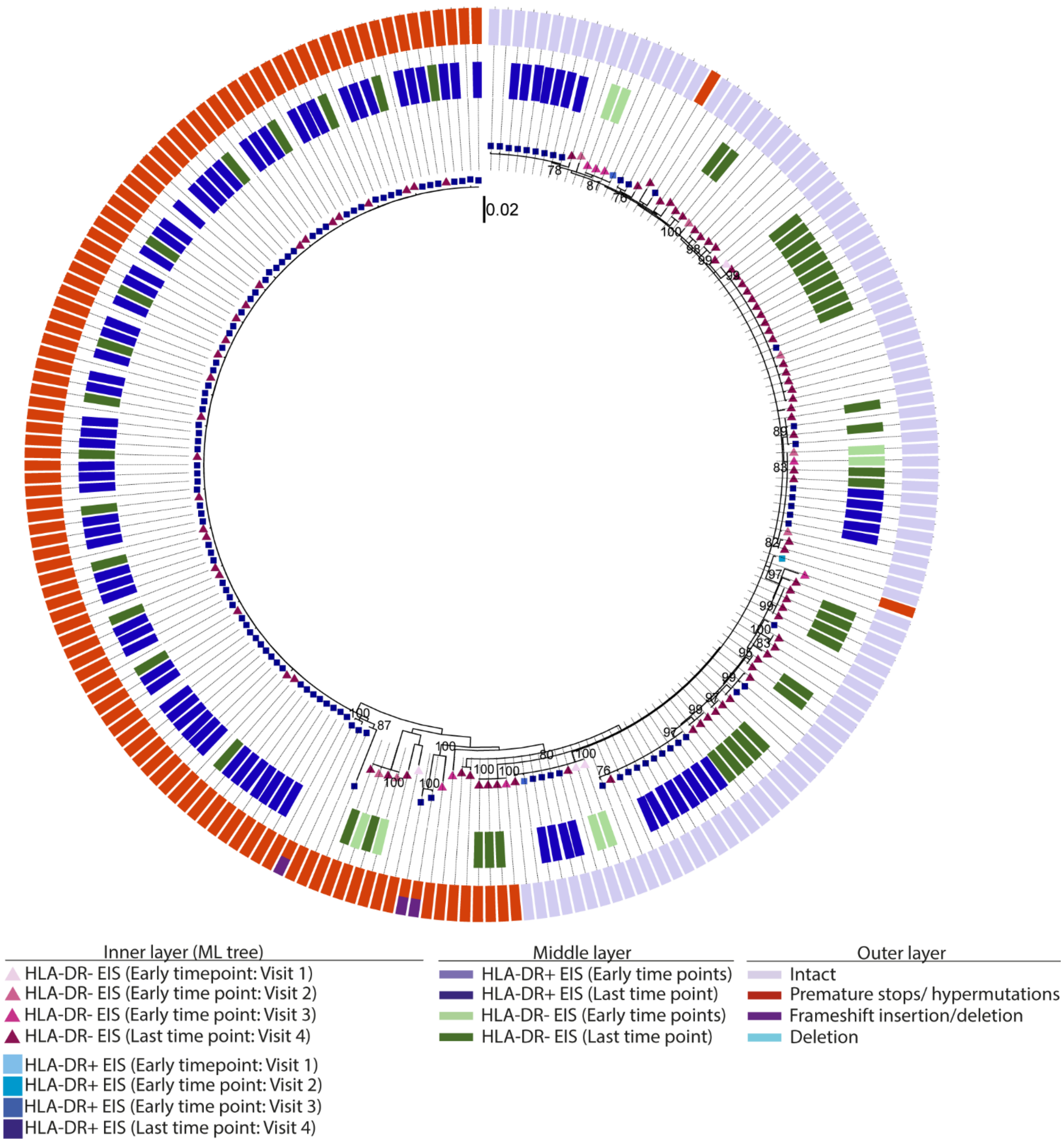

Supplementary figure 6. Maximum likelihood tree for participant 2013. The inner layer shows the phylogenetic tree, the middle layer shows individual HIV-DNA sequences which are part of expansions of identical HIV-DNA sequences (EIS) and the outer layer shows defective genetic features for individual HIV-DNA p6-RT sequences.

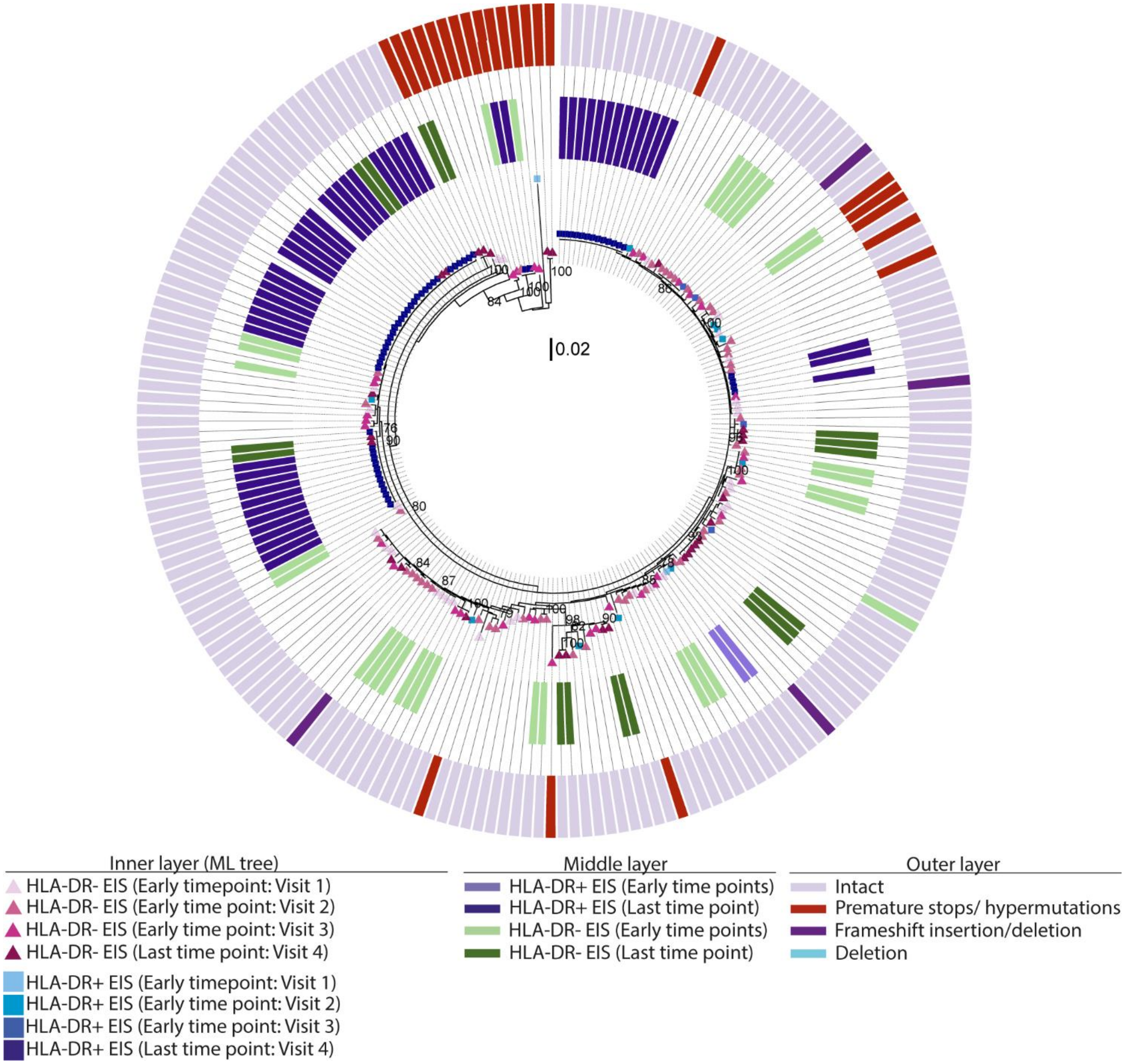

Supplementary figure 7. Maximum likelihood tree for participant 2046. The inner layer shows the phylogenetic tree, the middle layer shows individual HIV-DNA sequences which are part of expansions of identical HIV-DNA sequences (EIS) and the outer layer shows defective genetic features for individual HIV-DNA p6-RT sequences.

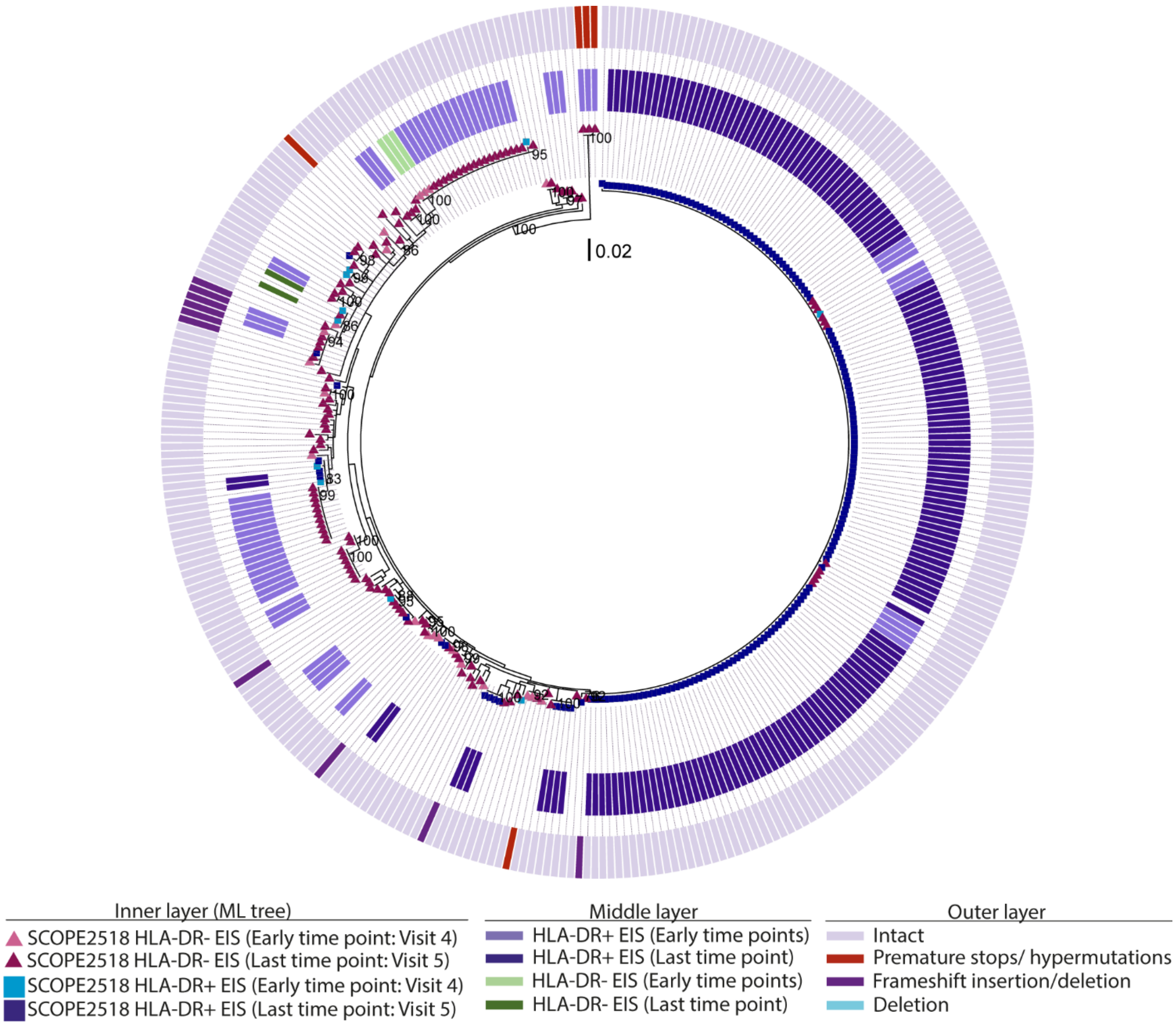

Supplementary figure 8. Maximum likelihood tree for participant 2518. The inner layer shows the phylogenetic tree, the middle layer shows individual HIV-DNA sequences which are part of expansions of identical HIV-DNA sequences (EIS) and the outer layer shows defective genetic features for individual HIV-DNA p6-RT sequences.

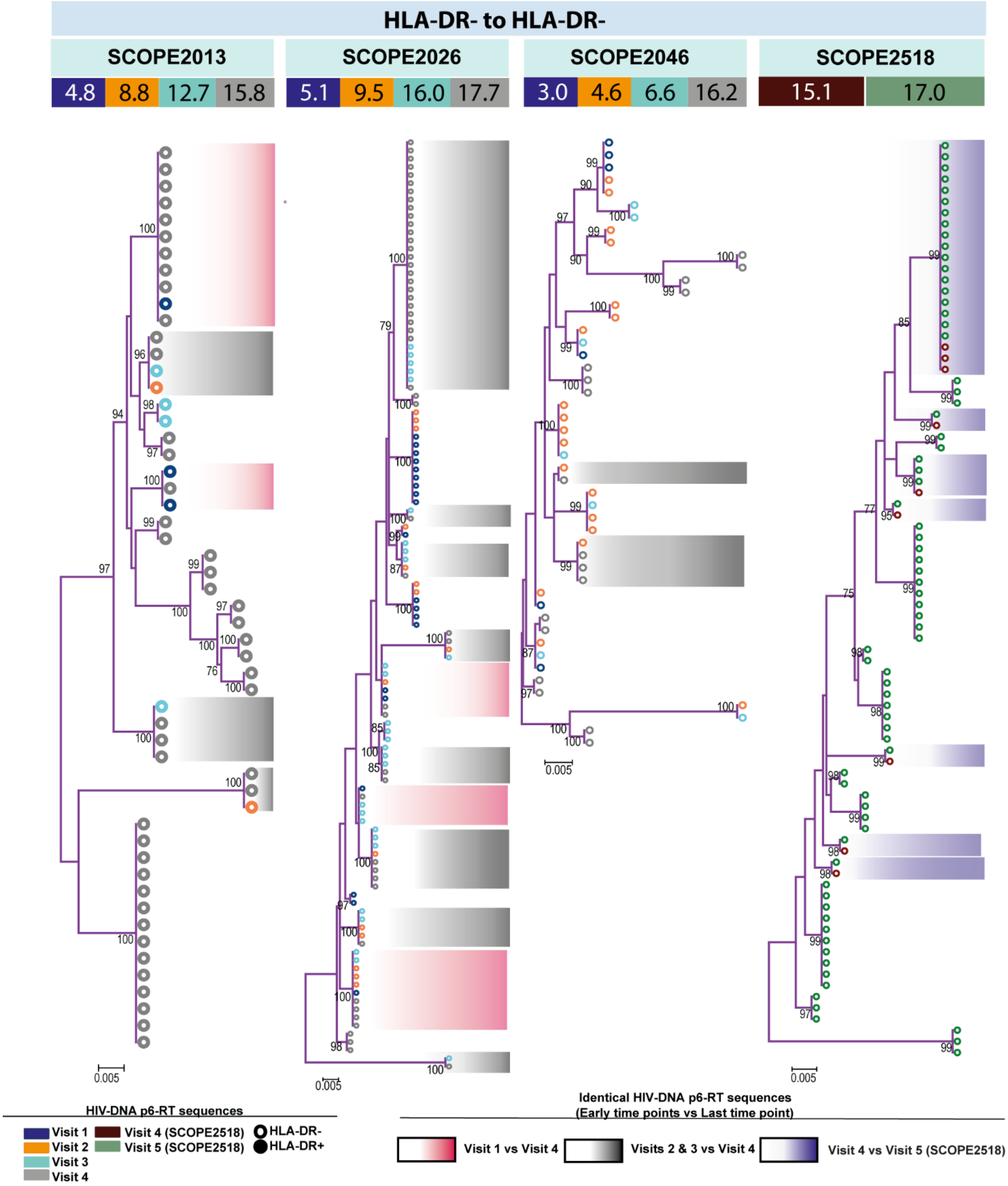

Supplementary figure 9. Persisting HIV-DNA p6-RT sequences within HLA-DR- CD4+ memory T-cell subset during ART.

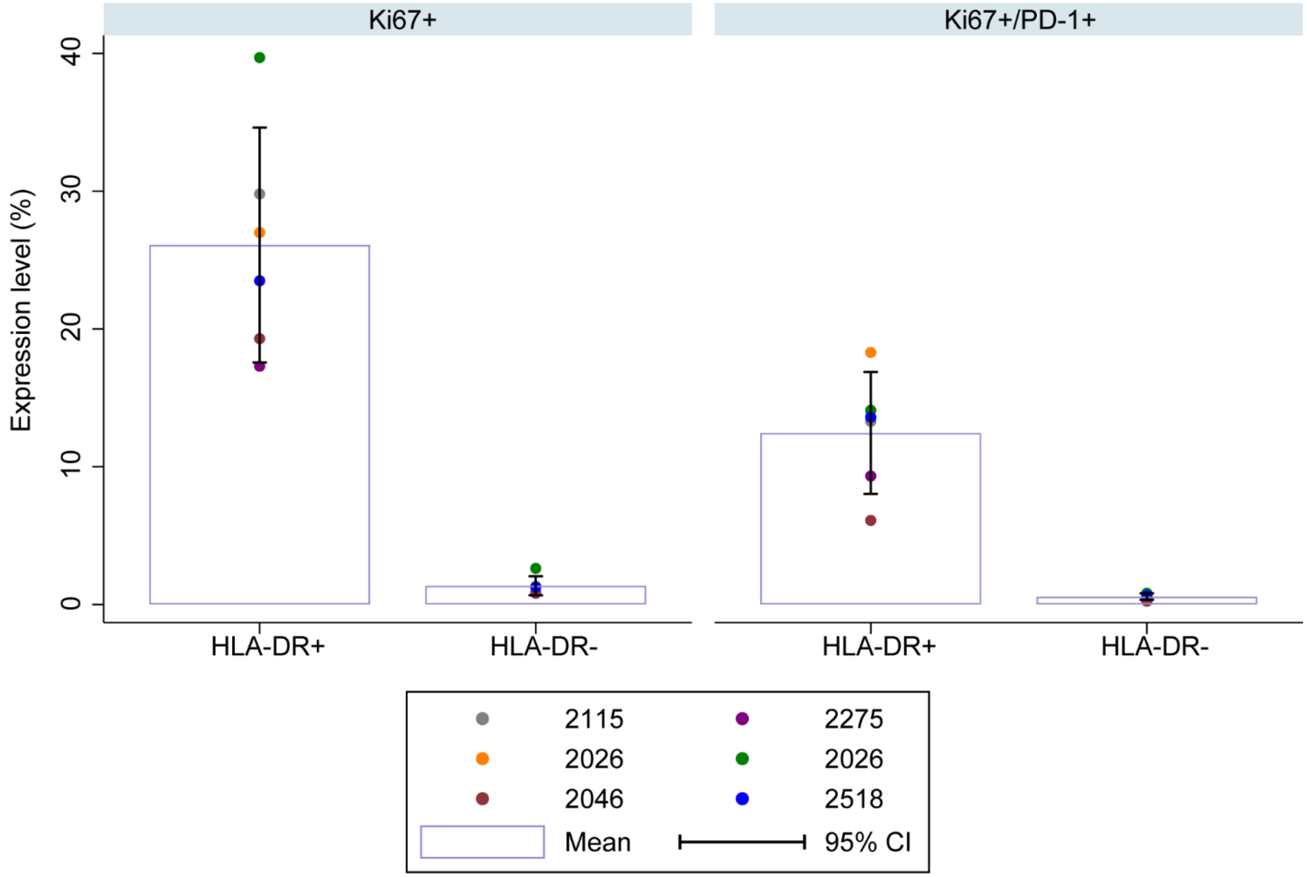

Supplementary figure 10. Expression levels of Ki67 and combined with PD-1 within HLA-DR+ and HLA-DR- CD4+ memory T-cell subsets during 15.1-17.7 years of ART (Visit ID 4 time points).

2 Supplementary tables

Supplementary table 1. Participant demographics.

Pt ID[A]	Pt Group[B]	Gender[C]	EID to ART start (m)[D]	Visit ID	ART start date	Years on ART[E]	CD4 count date	CD4 count	VL date	Viral load (copies per mL)	Viral load assay	Therapeutic Regimen	Sample type
2013	CHI	M	>12	1	1-May-96	4.8	5-Feb-01	640	5-Feb-01	<50	bDNA	ABC, 3TC, IDV	Stored PBMC
2013	CHI	M	>12	2	1-May-96	8.8	14-Feb-05	850	14-Feb-05	<75	bDNA	AZT, TDF, ABC/3TC	Stored PBMC
2013	CHI	M	>12	3	1-May-96	12.7	14-Jan-09	393	28-Jan-09	<40	Abbott Real Time PCR	ABC/3TC, ATV	Stored PBMC
2013	CHI	M	>12	4	1-May-96	15.8	28-Feb-12	1007	28-Feb-12	<40	Abbott Real Time PCR	ABC/3TC, ATV	Leukapheresis
2026	CHI	M	>12	1	1-Jan-96	5.1	18-Jan-01	546	18-Jan-01	<50	bDNA	ABC, D4T, NFV	Stored PBMC
2026	CHI	M	>12	2	1-Jan-96	9.5	20-Jun-05	588	11-Apr-05	<75	bDNA	ABC, TDF, NFV	Stored PBMC
2026	CHI	M	>12	3	1-Jan-96	16.0	5-Jan-12	617	4-Apr-12	<40	Abbott Real Time PCR	TDF, ABC/3TC, RTV, DRV	Stored PBMC
2026	CHI	M	>12	4	1-Jan-96	17.7	24-Sep-13	476	24-Sep-13	<40	Abbott Real Time PCR	TDF, ABC/3TC, RTV, DRV	Leukapheresis
2046	CHI	M	>12	1	27-Nov-97	3.0	27-Nov-00	534	27-Nov-00	<50	bDNA	D4T, 3TC, EFV, RTV, FTV	Stored PBMC
2046	CHI	M	>12	2	27-Nov-97	4.6	1-Jul-02	506	1-Jul-02	<50	bDNA	ABC, 3TC, EFV, LPV/r	Stored PBMC
2046	CHI	M	>12	3	27-Nov-97	6.6	21-Jun-04	705	21-Jun-04	<75	bDNA	ABC, 3TC, EFV, LPV/r	Stored PBMC
2046	CHI	M	>12	4	27-Nov-97	16.2	10-Feb-14	1099	10-Feb-14	<40	Abbott Real Time PCR	ECV, EFV/TDF/FTC	Leukapheresis
2518	CHI	F	>12	4	24-Jul-98	15.1	20-Sep-13	462	20-Sep-13	<40	Abbott Real Time PCR	TDF, AZT/3TC, NVP	Leukapheresis
2518	CHI	F	>12	5	24-Jul-98	17.0	N/A	N/A	N/A	N/A	N/A	TDF, AZT/3TC, NVP	Leukapheresis
2275	AHI	M	<6	1	23-Jul-98	2.9	6-Jun-01	1000	6-Jun-01	58	bDNA	NVP, AZT/3TC	Stored PBMC
2275	AHI	M	<6	2	23-Jul-98	4.7	9-Apr-03	1470	7-Jul-03	<75	Bayer HIV-1 RNA Assay	NVP, AZT/3TC	Stored PBMC
2275	AHI	M	<6	3	23-Jul-98	9.8	30-Apr-08	1950	30-Apr-08	<75	Bayer HIV-1 RNA Assay	FTC/TDF, NVP	Stored PBMC
2275	AHI	M	<6	4	23-Jul-98	15.1	17-Oct-13	1180	17-Oct-13	<40	Abbott Real Time PCR	FTC/TDF, NVP	Leukapheresis
2115	AHI	M	<6	1	20-Aug-96	4.6	27-Mar-01	741	27-Mar-01	<50	bDNA and Roche PCR	EFV, AZT/3TC	Stored PBMC
2115	AHI	M	<6	2	20-Aug-96	9.7	18-Apr-06	756	18-Apr-06	<75	bDNA	ABC/3TC, NVP	Stored PBMC
2115	AHI	M	<6	3	20-Aug-96	14.7	24-Jun-11	598	24-Jun-11	<40	Abbott Real Time PCR	FTC/TDF, NVP	Stored PBMC
2115	AHI	M	<6	4	20-Aug-96	17.2	12-Nov-13	601	12-Nov-13	<40	Abbott Real Time PCR	FTC/TDF, NVP	Leukapheresis

Abbreviations: [A]Pt ID, Participant ID;[B]Pt Group, Participant Group, CHI, Chronic, AHI, Acute/early; [C]M, Male, F, Female; [D]m, months; [E]Years on ART, Duration from ART start date to sample collection dates

Supplementary Table 2. Fluorochrome conjugated mouse and rat anti-human antibodies used for immunophenotyping human PBMC.

Name	Fluorochrome conjugate	Vendor	Clone
CD14	V500	BD	M5E2
CD3	Brilliant Violet 711	BD	UCHT-1
CD45RA	PE-Cy7	BD	L48
CD8	Brilliant Violet 605	BD	SK1
CD4	APC-eFluor780	eBioscience	OKT-4
HLA-DR	Brilliant Violet 421	BioLegend	L243
PD-1	Alexa 647	BD	EH12.1
TIM-3	PerCP-Cy5.5	BioLegend	F38-2E2
LAG-3	FITC	R&D Systems	Polyclonal
CD38	PE	BD	HB7
CCR5	PE-Cy5	BD	2D7
Dead Cell Marker	Aqua Amine Reactive Dye	Invitrogen	

Supplementary table 3. Number of CD4+ memory T-cells and HIV-DNA p6-RT sequences used for analysis (HLA-DR+).

PT[A]	PT group[B]	Visit ID	Years on ART	Cell number	Total number of HIV-DNA sequences	Number of defective HIV-DNA sequences	Number of HIV-DNA sequences from EIS
2115	AHI	1	4.6	65,455	2	0	NA
		2	9.7	99,545	10	4	NA
		3	14.7	96,818	2	0	NA
		4	17.2	327,781	38	7	NA
		Overall	4.6-17.2	589,599	52	11	NA
2275	AHI	1	2.9	156,818	4	0	NA
		2	4.7	120,000	1	0	NA
		3	9.8	199,091	21	0	NA
		4	15.1	505,709	65	5	NA
		Overall	2.9-15.1	981,618	91	5	NA
2013	CHI	1	4.8	105,000	2	0	0
		2	8.8	84,545	1	0	0
		3	12.7	132,273	0	0	0
		4	15.8	2,700,113	115	76	84
		Overall	4.8-15.8	3,021,931	118	76	84
2026	CHI	1	5.1	106,364	10	2	5
		2	9.5	148,636	1	0	0
		3	16.0	192,273	7	0	4
		4	17.7	244,091	65	0	51
		Overall	5.1-17.7	691,364	83	2	60
2046	CHI	1	3.0	122,727	2	1	1
		2	4.6	163,167	10	3	1
		3	6.6	133,636	4	1	0
		4	16.2	58,333	60	2	54
		Overall	3.0-16.2	477,863	76	7	56
2518	CHI	4	15.1	99,444	14	0	2
		5	17.2	92,728	184	2	167
		Overall	15.1-17.2	192,172	198	2	169
All participants (Total)				5,954,547	618	103	369

[A]PT, Participant; [B]PT group, participant group, AHI, participants who initiated ART during acute/early infection, CHI, participants who initiated ART during chronic infection

Supplementary table 4. Number of CD4+ memory T-cells and HIV-DNA p6-RT sequences used for analysis (HLA-DR-).

PT	PT group	Visit ID	Years on ART	Cell number	Total number of HIV-DNA sequences	Number of defective HIV-DNA sequences	Number of HIV-DNA sequences from EIS
2115	AHI	1	4.6	247,879	25	7	NA
		2	9.7	341,818	34	16	NA
		3	14.7	346,060	30	9	NA
		4	17.2	706,311	35	18	NA
		Overall	4.6-17.2	1,642,069	124	50	NA
2275	AHI	1	2.9	330,000	25	3	NA
		2	4.7	428,182	11	0	NA
		3	9.8	351,818	15	0	NA
		4	15.1	4,129,383	39	7	NA
		Overall	2.9-15.1	5,239,383	90	10	NA
2013	CHI	1	4.8	176,482	8	4	3
		2	8.8	297,273	7	2	1
		3	12.7	439,091	4	1	2
		4	15.8	4,187,364	87	34	47
		Overall	4.8-15.8	5,100,210	106	41	53
2026	CHI	1	5.1	167,728	41	8	20
		2	9.5	317,727	34	6	15
		3	16.0	695,455	51	5	27
		4	17.7	3,253,867	76	9	45
		Overall	5.1-17.7	4,434,776	202	28	107
2046	CHI	1	3.0	118,636	29	5	6
		2	4.6	135,000	54	6	17
		3	6.6	180,000	35	6	7
		4	16.2	163,889	30	5	16
		Overall	3.0-16.2	597,525	148	22	46
2518	CHI	4	15.1	455,309	20	3	3
		5	17.2	396,818	142	9	68
		Overall	15.1-17.2	852,127	162	12	71
All participants (Total)				$17.9X10^6$	832	163	277

[A]PT, Participant; [B]PT group, participant group, AHI, participants who initiated ART during acute/early infection, CHI, participants who initiated ART during chronic infection

Supplementary table 5. HIV-DNA levels measured by qPCR targeting LTR.

Pt ID[A]	Pt Group[B]	Years on ART	HLA-DR+		HLA-DR-	
			Sign for VL[C]	VL[D] (HIV-DNA copies/million cells)	Sign for VL[C]	VL[D] (HIV-DNA copies/million cells)
2115	AHI	4.6	<	1333.3	<	21.3
2115	AHI	9.7	<	418.4	<	16.4
2115	AHI	14.7	=	217.4	<	20.7
2115	AHI	17.2	=	32.7	=	41.1
2275	AHI	2.9	<	549.5	<	70.3
2275	AHI	4.7	<	430.1	<	72.3
2275	AHI	9.8	<	368.3	=	4.2
2275	AHI	15.1	=	242.6	=	133.9
2013	CHI	4.8	=	101.8	<	133.9
2013	CHI	8.8	<	324.7	=	6.7
2013	CHI	12.7	<	136.8	=	8.8
2013	CHI	15.8	<	35.5	<	59.9
2026	CHI	5.1	=	15.4	=	3.5
2026	CHI	9.5	<	211.9	=	3.9
2026	CHI	16.3	<	185	<	47.2
2026	CHI	17.7	=	34.1	=	44.6
2046	CHI	3.0	=	139.5	=	32.8
2046	CHI	4.6	=	11.7	=	72.2
2046	CHI	6.6	=	19.6	=	43.1
2518	CHI	15.1	=	83.7	=	2567.7
2518	CHI	17.0	=	529.9	=	7.9

[A]Pt ID, Participant ID; [B]Pt Group, Participant group, AHI, participants who initiated ART during acute/early infection, CHI, participants who initiated ART during chronic infection; [C]VL, Viral load, “<” VL estimated from limit of detection (1 HIV-DNA copy in an assayed sample), “=”, absolute value measured; [D]VL, Viral load

Supplementary table 6. Absolute number of HIV-DNA p6-RT sequences that indicate cellular persistence within HLA-DR+ or HLA-DR- CD4+ memory T-cell subset and cellular transition between HLA-DR+ and HLA-DR- phenotypes from earlier time points to last time point during ART.

Pt ID[A]	Pt Group[B]	Time point	Duration on ART (Years)[C]	Sample type[D]	Persistence[E]		Transition[F]	
					HLA-DR+ to HLA-DR+	HLA-DR- to HLA-DR-	HLA-DR+ to HLA-DR-	HLA-DR- to HLA-DR+
2115	AHI	Early vs Last	4.6-14.7 vs 17.2	Frozen PBMC vs Leukapheresis	8	0	0	30
2275	AHI	Early vs Last	2.9-9.8 vs 15.1	Frozen PBMC vs Leukapheresis	24	41	24	36
**Total (AHI group)**					32	41	24	66
2013	CHI	Early vs Last	4.8-12.7 vs 15.8	Frozen PBMC vs Leukapheresis	0	7	0	5
2026	CHI	Early vs Last	5.1-16.0 vs 17.7	Frozen PBMC vs Leukapheresis	3	39	6	12
2046	CHI	Early vs Last	3.0-6.6 vs 16.2	Frozen PBMC vs Leukapheresis	0	2	1	3
2518	CHI	Early vs Last	15.1 vs 17.0	Leukapheresis vs Leukapheresis	3	9	4	2
**Total (CHI group)**					6	57	11	22
**Total (all participants)**					38	98	35	88

[A]Pt ID, Participant ID; [B]Pt Group, participants who initiated ART during acute/early (AHI) and chronic infection (CHI); [C]Duration on ART of earlier time points (Early) and last time point (Last); [D]Sample types that HLA-DR+ and HLA-DR- CD4+ memory T-cells were sorted from; [E]Persistence, HIV-DNA p6-RT sequences obtained at earlier time points that were genetically identical to at least one viral sequences obtained at last time point within HLA-DR+ (HLA-DR+ to HLA-DR+) or HLA-DR- CD4+ memory T-cell subset (HLA-DR- to HLA-DR-); [F]Transition, HIV-DNA p6-RT sequences derived from the HLA-DR+ T-cell subset at earlier time points that were genetically identical to at least one viral sequences derived from the HLA-DR- T-cell subset at last time point (HLA-DR+ to HLA-DR-). HIV-DNA p6-RT sequences derived from the HLA-DR- T-cell subset at earlier time points that were genetically identical to at least one viral sequence derived from the HLA-DR+ T-cell subset at last time point (HLA-DR- to HLA-DR+)

Supplementary table 7. Correlation between proportions of HIV-infected CD4+ memory T-cells and genetically identical HIV-DNA sequences from EIS and expression levels of cellular proliferation markers.

Comparisons[A]	N[B]	Spearman Rank Correlation (ρ)	95% CI		P-value
			Lower	Upper	
**HLA-DR+**					
%Infected with %EIS	4	1.00	.	1.00	0.083
%Infected with Ki67+	6	-0.143	-0.855	0.756	0.79
%Infected with Ki67+/PD-1+	6	-0.486	-0.930	0.538	0.33
%EIS with Ki67+	4	-.200	-.974	0.942	0.80
%EIS with Ki67+/PD-1+	4	-.400	-.983	0.912	0.60
**HLA-DR-**					
%Infected with %EIS	4	-0.800	-0.996	0.697	0.20
%Infected with Ki67+	6	0.086	-0.780	0.839	0.87
%Infected with Ki67+/PD-1+	6	-0.143	-0.855	0.756	0.79
%EIS with Ki67+	4	0.400	-.912	0.983	0.60
%EIS with Ki67+/PD-1+	4	0.400	-.912	0.983	0.60

[A]Ki67 and Ki67/PD1 were measured within total CD4+ T-cells derived from peripheral blood obtained from Visit ID 4 (see Supplementary Table 1). EIS (expansions of genetically identical HIV-DNA sequences) was measured within participants from the CHI group, %Infected, proportion of HIV-1 infected CD4+ memory T-cells; [B]N, number of participants included in the correlation.

Supplementary table 8. Odds ratio of an HIV-DNA p6-RT sequence being a part of EIS.

Comparisons[A]	Included Participant	Time points (yrs)[B]	OR[C]	95% CI		P-value
				Lower	Upper	
**Early time point(s)**						
HLA-DR+ vs HLA-DR-	2013	4.8-12.7	0.608	0.285	1.298	0.199
	2026	5.1-16.0				
	2046	3.0-6.6				
HLA-DR+ vs HLA-DR-	2518	15.1	1.26	0.18	8.97	0.818
**Last time point**						
HLA-DR+ vs HLA-DR-	2013	15.8	3.14	2.02	4.90	<0.001
	2026	17.7				
	2046	16.2				
HLA-DR+ vs HLA-DR-	2518	17.0	18.7	8.5	41.2	<0.001

[A]HLA-DR+, HLA-DR+ CD4+ memory T-cells, HLA-DR- HLA-DR- CD4+ memory T-cells; [B]Years on therapy at clinical sample collection; [C]OR, Odds ratio
